# A novel STK1-targeted small-molecule as an “antibiotic resistance breaker” against multidrug-resistant *Staphylococcus aureus*

**DOI:** 10.1038/s41598-017-05314-z

**Published:** 2017-07-11

**Authors:** Sashi Kant, Shailendra Asthana, Dominique Missiakas, Vijay Pancholi

**Affiliations:** 1 Department of Pathology, The Ohio State University College of Medicine, Columbus, Ohio USA; 2 Drug Discovery Research Center, Translational Health Science and Technology Institute, Faridabad-Gurgaon Expressway, Haryana, India; 3 Department of Microbiology, University of Chicago, Chicago, Illinois USA

## Abstract

Ser/Thr protein kinase (STK1) plays a critical role in cell wall biosynthesis of and drug resistance in methicillin-resistant *Staphylococcus aureus* (MRSA). MRSA strains lacking STK1 become susceptible to failing cephalosporins, such as Ceftriaxone and Cefotaxime. STK1, despite being nonessential protein for *MRSA* survival, it can serve as an important therapeutic agent for combination therapy. Here, we report a novel small molecule quinazoline compound, Inh2-B1, which specifically inhibits STK1 activity by directly binding to its ATP-binding catalytic domain. Functional analyses encompassing *in vitro* growth inhibition of MRSA, and *in vivo* protection studies in mice against the lethal MRSA challenge indicated that at high concentration neither Inh2-B1 nor Ceftriaxone or Cefotaxime alone was able to inhibit the growth of bacteria or protect the challenged mice. However, the growth of MRSA was inhibited, and a significant protection in mice against the bacterial challenge was observed at a micromolar concentration of Ceftriaxone or Cefotaxime in the presence of Inh2-B1. Cell-dependent minimal to no toxicity of Inh2-B1, and its abilities to down-regulate cell wall hydrolase genes and disrupt the biofilm formation of MRSA clearly indicated that Inh2-B1 serves as a therapeutically important “antibiotic-resistance-breaker,” which enhances the bactericidal activity of Ceftriaxone/Cefotaxime against highly pathogenic MRSA infection.

## Introduction

Highly pathogenic and multidrug-resistant *S*. *aureus* (MDRSA), including methicillin, vancomycin-, daptomycin- and linezolid-resistant *S*. *aureus* are continuously replacing the traditional methicillin-resistant *S*. *aureus* (MRSA) in the community as well as in the hospital^1–4^. With a lag in the development of new, broad-spectrum antibiotics from pharmaceutical companies^5, 6^, the emergence of multidrug-resistant traits in highly pathogenic community-associated *S*. *aureus* strains^7^ demands identification of novel chemotherapeutic agents for the effective control of MRSA/MDRSA dissemination.

Two-component regulatory systems (TCSs) constituted by sensor histidine kinases (HK), and response regulators (RR) allow bacteria to respond rapidly to environmental changes by modulating the transcription of genes in a coordinated manner^8^. *S*. *aureus* encodes several TCSs that control a variety of metabolic functions, cell division/cell wall biosynthesis, virulence, and multiple drug resistance^9, 10^ through His and Asp residue phosphorylation mechanisms^8, 11^. Eukaryote-type Ser/Thr protein kinases (STKs) and phosphatases (STPs) are conserved in several Gram-positive bacteria^12^. They provide an additional level of regulation for a variety of biological functions, including, metabolic regulation and fitness, cell wall biosynthesis, cell division, resistance to an antimicrobial peptide, expression of virulence factors, virulence regulation, biofilm formation, antibiotic efflux functions, and drug resistance^12^. This regulation occurs via post-translational modifications mediated by the reversible phosphorylation of certain Ser/Thr residues of the targeted proteins^13^. In *S*. *aureus*, STK1 and STP1 modulate the activity of several TCSs and stand-alone or mono-component regulators^14–19^. They have also been incriminated in the reciprocal modulation of susceptibility to cell wall acting antibiotics such as certain cephalosporins^20^ and vancomycin^16, 21, 22^. *S*. *aureus* STK1-dependent vancomycin resistance has been attributed to the Thr-phosphorylation of VraR (T106, T119, T175, T178)^16^ and GraR (T128, T130) TCS regulators^19^. Quinolone resistance has been attributed to STK1-dependent phosphorylation of the stand-alone regulator MgrA at Ser110 and Ser113. Phosphorylation affects the DNA binding activity of MgrA resulting in derepression of *norA* transcription, a gene that encodes the efflux pump responsible for quinolone efflux^17, 23^. STK1 and STP1 have also been proposed to modify Thr residues of SarA^14^ and CcpA^15^ as well as Cys residues of MgrA, SarA, SarZ, and CymR regulators^18^. Thus, eukaryote-type STK and STP enzymes contribute broadly to the expression of genes involved in virulence and antibiotic resistance. The deletion or acquisition of naturally occurring point mutations in the *stp1* gene under selective pressure results in decreased susceptibility to many important antibiotics^21, 22, 24, 25^. Paradoxically, naturally occurring mutations in the *stk1* gene have not been observed so far. STK1 as well as STP1 are not essential for *S*. *aureus* ^20^. Cumulative findings suggest that STK1 may serve as a better target than STP1 for the development of therapeutics against multi-drug-resistant *S*. *aureus*. Thus, the inhibition of STK1 activity by an inhibitor may help reduce the *S*. *aureus* resistance against cell wall acting antibiotics.

In the present investigation, we test a hypothesis that STK1 serves as a novel target for the development of a small molecule-based therapeutic agent by acting as an “antibiotic resistance breaker”. We further test that such an agent can potentiate the bactericidal activity of the cell wall acting antibiotics which once served as” life-saving drugs” are now deemed to be “off the shelf” or the failing antibiotics due to the emergence of multidrug-resistant bacteria. We provide a proof for this hypothesis by identifying a small molecule inhibitor (Inh2-B1) that specifically targets STK1, alters cell wall biosynthesis, and adversely affects biofilm formation of *S*. *aureus*. Further, using the mouse model of *S*. *aureus* septicemia, we confirm that the compound, Inh2-B1, potentiates the bactericidal activity of cell-wall acting cephalosporins, Ceftriaxone and Cefotaxime, and provides significant protection against lethal MRSA infection.

## Results

### STK1 and STP1 reciprocally regulate the growth in S. aureus MW2 strain

**Figure 1:**
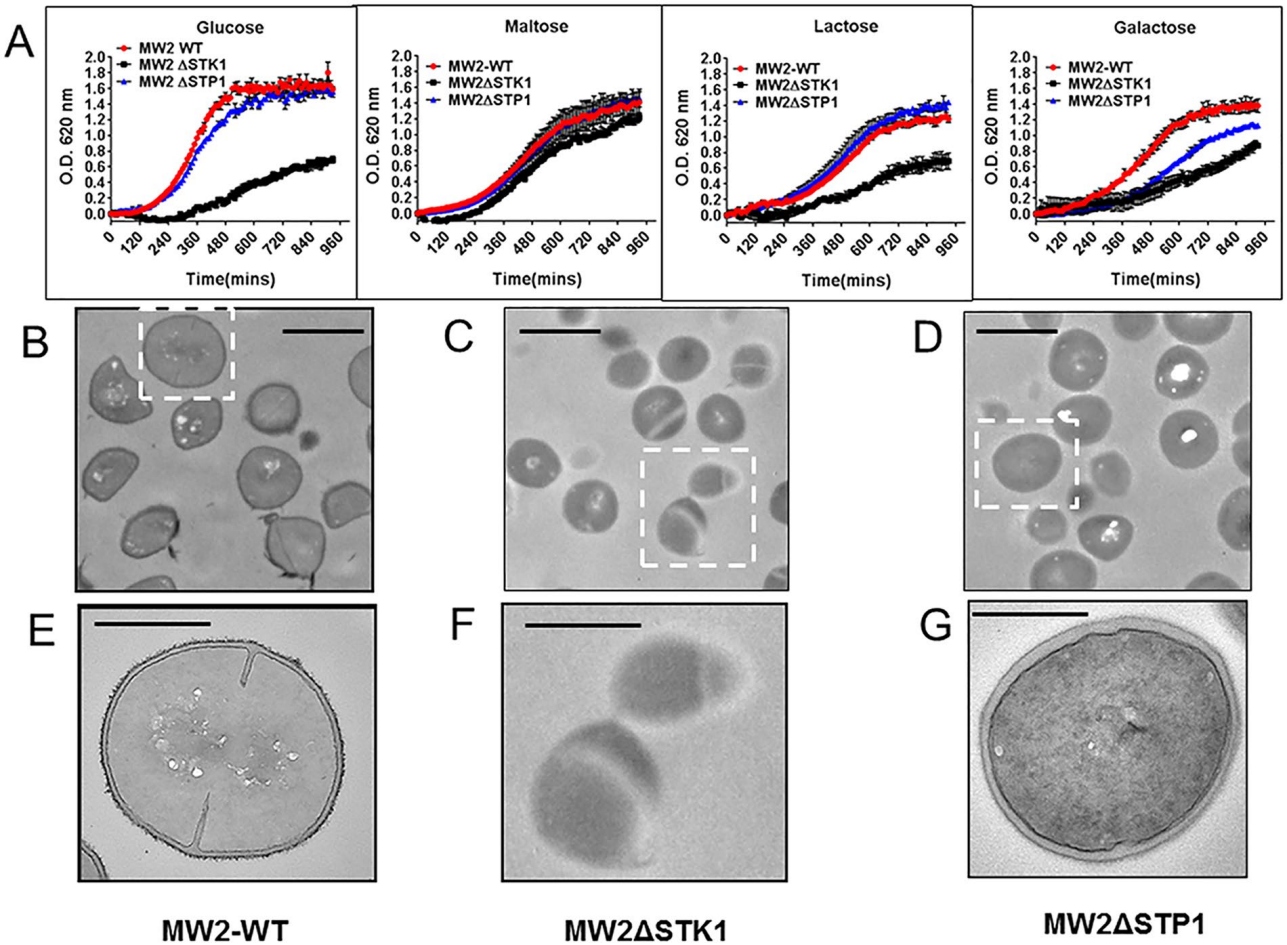
The impact of the deletion of *stk1* and *stp1* genes in *S*. *aureus* MW2 growth and cell wall structure. (**A**) Growth patterns of the *S*. *aureus* MW2-wild-type, MW2ΔSTK1, and MW2ΔSTP1 strains in Chemically defined medium (CDM) supplemented with 1% carbohydrate (glucose, maltose, lactose, or galactose). Growth kinetics were measured in 96-well plates at 37 °C for 16 hours under constant rotation in a final volume of 200 µl using sterile 96-well plates. Optical density at 620 nm was measured at every 15 min. Data at each time point represent an O.D. value (mean ± SD) obtained from three different experiments and each with quadruple wells for each strain. O.D. was measured spectrophotometrically as described in the Materials and Methods. Growth curves are shown in Red – MW2-wild-type strain; Black – MW2ΔSTK1 mutant; Blue – MW2ΔSTP1 mutant strains. (**B**–**D**) Transmission electron microscopy (TEM) at a low resolution of (**B**) wild-type MW2 and corresponding isogenic (**C**) ΔSTK1 and (**D**) ΔSTP1 mutants. The wild-type and mutant strains were grown in TSB, fixed in the glutaraldehyde and paraformaldehyde fixative and subsequently subjected to transmission electron microscopy as described in Materials and Methods. Inset bar-1.0 µm. (**E**–**G**) TEM of (**B**–**D**) at a higher resolution. Inset bar-500 nm.

Previously, we and others have reported that the growth of *S*. *aureus* isogenic mutants lacking STK1, but not STP1, is retarded when compared to the parent wild-type strains^20, 26^. Considering the wide range of prevailing strain variations in MRSA for virulence as well as drug resistance, we derived ΔSTK1 and ΔSTP1 mutants from a community-associated and highly pathogenic multidrug-resistant *S*. *aureus* strain (MW2) in the present investigation (Fig. 1). We further investigated the impact of deletion of these genes on the growth as well as the susceptibility of the mutants against cell wall acting antibiotics. In comparison to the Wild-type strain, MW2ΔSTK1 mutant showed colonies with a larger hemolytic zone (MW-WT) on blood agar plates. On the other hand, the MW2ΔSTP1 mutant strain developed non-hemolytic colonies (Fig. S1). These results concurred with those reported for similar mutants derived from *S*. *aureus* Newman strain^27^. The growth of MW2ΔSTK1 and not MW2ΔSTP1 was retarded in chemically defined medium (CDM) supplemented with different carbohydrate sources indicating that the STK1 activity contributes to overall staphylococcal metabolic fitness (Fig. 1A). Electron microscopy of the MW2ΔSTK1 mutant in comparison to the MW2-wildtype (Fig. 1B and E) revealed altered cell wall structures and defective cell septa in ~40% of cells (Fig. 1C and F). These features were in contrast to the MW2ΔSTP1 mutant displaying intact and thick cell wall phenotypes (Fig. 1D and G), as reported previously for *S*. *aureus* N315 and USA300^20, 22^.

*S*. *aureus* STK1 has become an attractive target because of its close association with cell wall biosynthesis process^13, 14, 20, 28, 29^ and the observation revealing the increased susceptibility to cell wall acting antibiotics^14, 20, 29^. Initial screening of antibiotic susceptibility by E™-test revealed increased susceptibility of MW2ΔSTK1 mutant to Ceftriaxone (MIC = 4 µg/ml) and Cefotaxime (MIC = 4 µg/ml) in comparison to the MW2-WT (>100 µg/ml) and MW2ΔSTP1 mutant (>100 µg/ml) (Fig. S2A). These results confirmed our earlier findings with *S*. *aureus* N315 strains^20^, and thus indicated that the innate antibiotic resistance of *S*. *aureus* can be substantially reduced by inhibiting the STK1 activity (Fig. S2A).

### Identification of small molecule inhibitors of STK1

**Figure 2:**
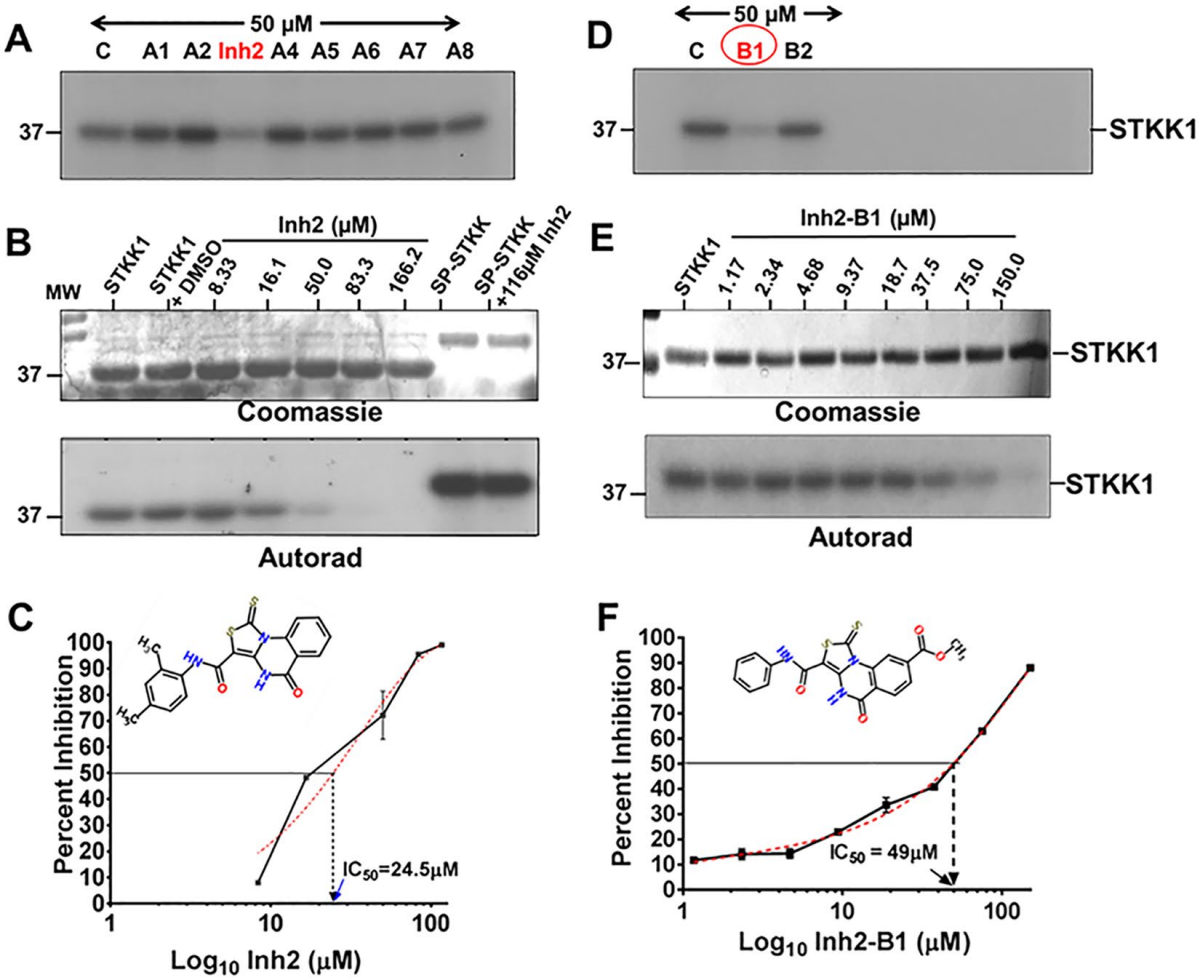
Identification of small molecule inhibitors targeted to *S*. *aureus* STK1. (**A**,**D**) Screening of Inh2-derivatives (50 µM as indicated) for their ability to inhibit the kinase activity of *S*. *aureus* STKK1. (**B**,**E**) Coomassie-stained gels and corresponding autoradiograph show the dose-dependent inhibitory activity of a small molecule inhibitors, (**B**) Inh2 (N-(2,4-Dimethylphenyl)-5-oxo-1-thioxo-4,5-dihydro [1,3] thiazolo[3,4-α] quinazoline-3-carboxamide) and (**E**) Inh2-B1 [Methyl 5-oxo-3-(phenyl carbamoyl)-1-thioxo-4,5dihydro[1,3]thiazolo[3,4-a]quinazoline-8-carboxylate] targeted to *S*. *aureus* STKK1 (the soluble kinase domain of STK1, 280 aa). (**C**,**F**) Quantitative analysis (IC_50_ concentration) of Inh2 and Inh2-B1-mediated inhibition of STKK1 activity after 5 min pre-incubation. Each data point represents an average percent inhibition of three individual readings of radioactivity incorporation (radioactive counts per minute or CPM) (Mean ± SEM) in protein bands of samples treated with different concentrations of the inhibitor in comparison to control samples. CPM measured in the protein band of the control sample without inhibitor was treated as 100% incorporation or 0% inhibition.

Based on the above results, we hypothesized that STK1 could serve as a novel therapeutic target, and a putative inhibitor of STK1 serve as an “antibiotic resistance breaker”. Further, this inhibitor, in turn, could potentiate the bactericidal activity of some of the cell wall acting antibiotics, which are deemed “inactive” or “failing” due to the emergence of MDRSA. Quinazoline-based small molecule compounds have been shown to inhibit kinase activity^30–32^. Several amino-quinazoline-based inhibitors (Mitoxantrone, VI12112) have been described to inhibit the Ser/Thr kinase activity of mycobacterial PknB^33, 34^. In our recently prescreened library of 32 small molecule compounds that inhibited the growth of *S*. *aureus* RN4220 at varying concentrations ranging from 25–100 µM^35^, we found that the core structure of many of these compounds belonged to quinazoline group. We, therefore, predicted that one or more compounds of this prescreened library might possess STK1 inhibitory activity. Although STKs are transmembrane proteins, the isolated kinase domains of these proteins have been found to be soluble and when purified from *E*. *coli* retain enzymatic activity^20, 36–40^. Further, the crystal structure of the soluble STK1 kinase domain referred here as STKK1 has recently been solved^38^. We, therefore, used the purified recombinant STKK1 protein (first 280 aa of STK1)^20^ for further experimental purposes. Upon screening of these pre-screened compounds for their ability to inhibit the *in vitro* autophosphorylation activity of STKK1, we identified Inh2 (381.5 Da, Fig. 2A–C, Fig. S3A–C), Inh31 (293.4 Da, Fig. S4A) and Inh32 (293.4 Da, Fig. S4B) as three potential candidates. Maximum inhibition of the kinase activity of STKK1 was observed using Inh2 (IC_50_ 24.5 µM) (Fig. 2A–C, Fig. S3A–C) followed by Inh31 (60 µM) (Fig. S4A) and Inh32 (92.0 µM) (Fig. S4B). We, therefore, focused on Inh2, which is chemically characterized as N-(2,4-Dimethylphenyl)-5-oxo-1-thioxo-4,5-dihydro[1,3]thiazolo[3,4-a] quinazoline-3-carboxamide (Fig. 2C, Fig. S5). The Inh2 compound also appeared to be specific for staphylococcal STK1, since even at 116 µM concentration, it did not inhibit autophosphorylation of *S*. *pyogenes* SP-STK^36^ (Figs 2B, S3B,C). Although the MW2 mutant lacking STK1 displayed retarded growth (Fig. 1), STK1 as such is not essential for bacterial survival. However, in the broth dilution-based antibiotic susceptibility assays, the IC_50_ concentration of Inh2 for MW2 was found to be 12.0 µM and minimum inhibitory concentration of 48 µM indicating that Inh2 can have an additional off-target activity (Fig. S2B). Inh2 was, therefore, used as a lead compound to obtain relevant derivatives that show a comparable Ser/Thr kinase inhibitory activity and phenotypic characteristics of the MW2ΔSTK1 mutant, i.e., retarded growth but not bactericidal activity.

To validate and establish the physiologically relevant chemical inhibition of STK1 activity by Inh2, we synthesized ten derivatives adhering to the Lipinski RO5 rule^41^ using the on-line tool ZINC (http://zinc.docking.org) (Fig. S5). We then examined all ten derivatives for their ability to inhibit the kinase activity of STKK1 as well as growth inhibition of *S*. *aureus* MW2 as described above. Only Inh2-B1 [Methyl 5-oxo-3-(phenyl carbamoyl)-1-thioxo-4,5dihydro[1,3]thiazolo[3,4-a]quinazoline-8-carboxylate] inhibited kinase activity of STKK1 (IC_50_ = 49 µM) (Fig. 2D–F, and Fig. S3D–F) with growth even at concentration of >50 µM as shown below. Inh2-B1 did not show any changes in the hemolysis pattern of colonies of MW2 -WT, MW2ΔSTK1 or MW2ΔSTP1 (Fig. S1) indicating that increased hemolysis may not be the outcome of direct STK inhibition but by the factors modulated within the downstream signaling pathways which may also be influenced by other co-regulator^14^.

### Inh2 and Inh2-B1 compete with ATP for binding to the catalytic domain of STK1

**Figure 3:**
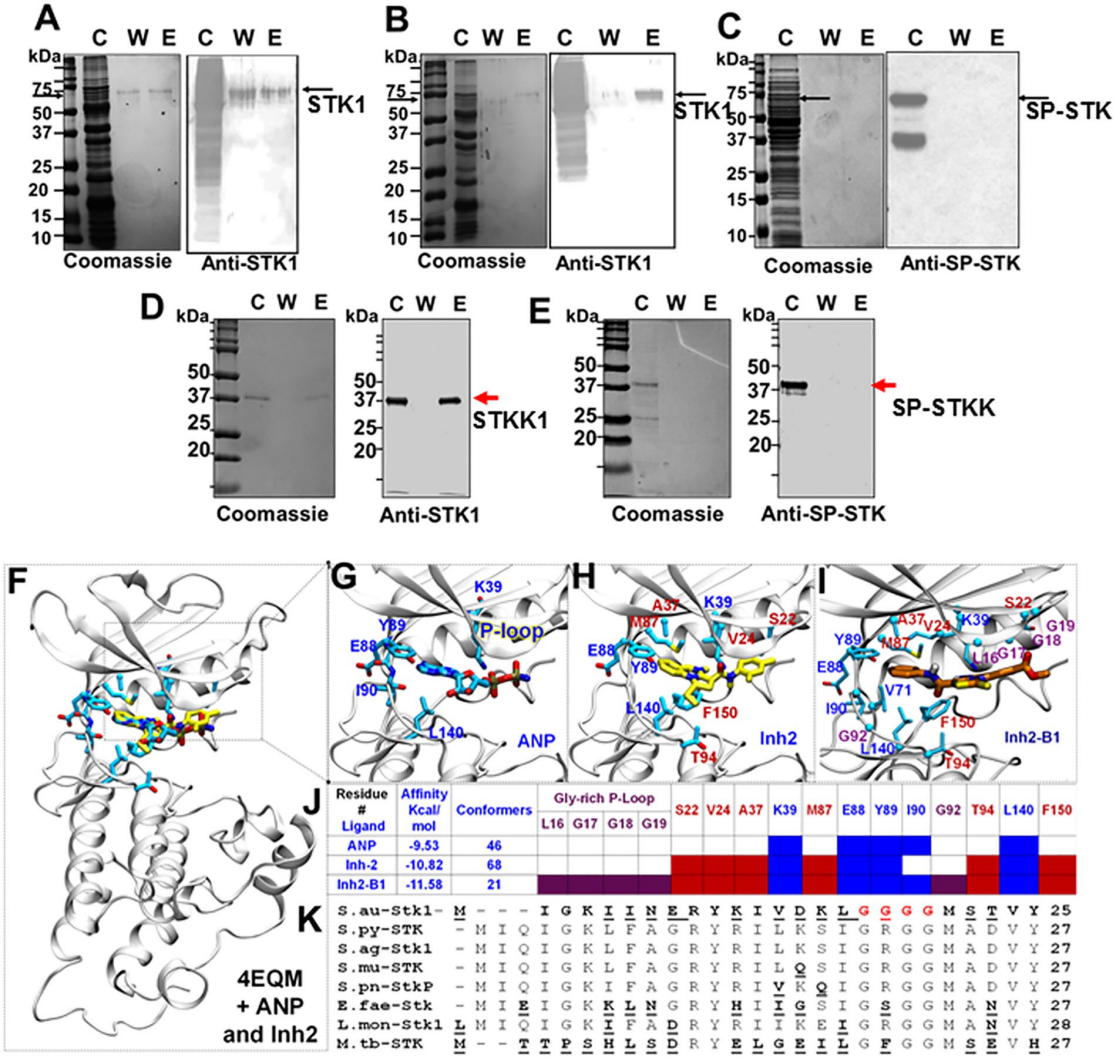
Binding of Inh2 and Inh2-B1 to the catalytic ATP-binding pocket of STK1. *In vivo* binding of (**A**) Inh2 and (**B**) Inh2-B1 to *S*. *aureus* STK1 as determined by ATP-column chromatography. Whole cell lysostaphin-digest of the MW2-wild type strain was passed on to the buffered equilibrated ATP-agarose column and eluted with 50 µM Inh2 or Inh2-B1 followed by detection of STK1 in Western Blot assay using the anti-STK1 polyclonal antibody as described in the Materials and Methods section. (**C**) A similar experiment with whole cell lysate of the phage-lysin digested *S*. *pyogenes* M1T1 strain revealing no elution of SP-STK. STK1 (~90 kDa) and SP-STK (~70 kDa) proteins (see pointed arrow) in their respective bacterial lysates are found without/ with its degraded forms as described previously^20, 36^. C-cell lysate, W-wash fraction, E- elution fraction. (**D**) Binding of the purified recombinant STKK1 to solid-phase ATP- column and its elution by Inh2-B1. (**E**) Binding of the purified recombinant SP-STK to solid-phase ATP- column and its elution by Inh2-B1. (**F**) *In silico*, molecular docking analysis-based the highest scored docked pose of ANP and Inh2 in the binding pocket of the kinase domain of *S*. *aureus* STK1 (PDB ID 4EQM). The protein is shown in the white cartoon. Interaction of (**G**) ANP (inactivated ATP shown in cyan), (**H**) small molecule compound Inh2 (shown in yellow) and (**I**) Inh2-B1 (shown in dark orange) and key residues (only the side-chain non-hydrogenous atoms) around 3.5 Å from the inhibitor are shown in the sticks. The coloring code of the atom type: C (Yellow or green in inhibitors and cyan in ANP), N (blue), and O (red). (**J**) A summary table is showing the association of ANP, Inh2, and Inh2-B1 with amino acid residues reflecting their affinity for the ATP-binding pocket and a number of conformers clustered in this region during molecular docking analysis. ANP constitutes key residues (blue fonts). The residues around Inh-2 are shown in red fonts and the residues around P-loop that directly interact with Inh2-B1 are shown in purple fonts (See also Fig. S5 for affinity and number of conformers for all Inh2 derivatives). (**K**) A comparison of the N-terminal sequence of STK1 with those of other Gram-positive pathogens showing the unique nature of the *S*. *aureus* STK1 P-loop (Red fonts). The underlined bold letters denote non-conserved amino acid residues.

To determine the *in vivo* binding ability of Inh2 and Inh2-B1 to STKK, the whole cell lysate of *S*. *aureus* MW2 was subjected to solid-phase ATP affinity chromatography. Bound proteins of the whole cell lysates to the ATP-column were eluted with 50 µM Inh2 and Inh2-B1 inhibitors, and subsequently resolved by SDS-PAGE, and visualized by Coomassie staining or electrotransferred to a PVDF membrane. Immunoblot analysis using an anti-STK1 antibody revealed the presence of STK1 in the Inh2- and Inh2-B1-eluted fractions (Fig. 3A and B). When the experiment was repeated with group A *Streptococcus* (*S*. *pyogenes*) whole cell lysates, Inh2 did not elute the bound SP-STK (Fig. 3C). Similar results were also obtained by using Inh2-B1 instead of Inh2 (data not shown). To confirm these findings, we subjected the purified recombinant *S*. *aureus* STKK1 protein and *S*. *pyogenes* SP-STKK protein to solid-phase-ATP-column chromatography as described above and eluted with Inh2-B1. The latter could elute only the *S*. *aureus* STKK1 protein (Fig. 3D) and not *S*. *pyogenes* SP-STKK protein (Fig. 3E). These results indicated that Inh2-B1 did not target *S*. *pyogenes* SP-STK *in vivo* as was observed by *in vitro* assays (Fig. 2A–F).

Further, to define the binding site of the inhibitor within the catalytic site of the STK1 protein, the small molecule inhibitors (Inh2 and all Inh2-derivatives including Inh2-B1) were subjected to molecular docking analysis. The latter was achieved by employing blind-docking on the available 3D structure of the kinase domain of STK1 (PDB 4EQM) (Fig. 3F, Fig. S6). By employing the Autodock grid (126 × 71 × 71) on this model and using the Lamarckian genetic algorithm, we examined 200 runs with 300 random individuals in the first population for each docking simulation. The lowest-energy conformation of each inhibitor was chosen, clustered, and ranked based on the energy score according to an RMSD cutoff of 3 Å. This cluster was found to localize in the ATP-binding pocket (Fig. 3F). Subsequently, narrow grids of dimensions 50 × 50 × 50 were employed in the region containing the inactive ATP analog, ANP. The focused or targeted docking was reiterated to identify clusters with the highest number of populated clusters (conformers) and lowest energy for Inh2, ANP, as well as nine Inh2-derivatives (Fig. S6). This series of simulation placed Inh2 in a position that is superimposable to the ATP binding site (Fig. 3G). In the 4EQM structure, five residues K39, E88, Y89, I90, and L140 were found to form an interaction network with bound ANP (inactive ATP) (Fig. 3G, see also Fig. 3J; residues depicted in blue)^38^. Our docking analysis identified six additional residues S22, V24, A37, M87, T94, and F150 in the proximity of Inh2 (Fig. 3H; residues depicted in red). In this model, Inh2 interacted with the protein at high affinity (−10.82 kcal mol^−1^ with 68 conformers (Fig. 3J and Fig. S6). Interestingly, among all ten derivatives, Inh2-B1 showed the highest theoretical affinity for the catalytic domain of the STKK1 (−11.58 kcal mol^−1^ with 21 conformers Fig. 3J and Fig. S6). Docking analysis also revealed the interaction of Inh2-B1compound with a glycine-rich loop (G17-G19) as well as additional interacting amino acid side chains of L16 and G92, forming a unique pose that is different from that of ANP and Inh2 (Fig. 3I). A comparison of sequences encompassing the catalytic domain of various Gram-positive STKKs also indicated that a motif containing G^17^GGG^20^ residues is uniquely present only in *S*. *aureus*, unlike GRGG in STKs of other Gram-positive pathogens (Fig. 3K). This comparison also concurs with the molecular docking-based predictive analysis of the affinity of Inh2-B1 for *S*. *aureus* STKK1 and supports the inability of Inh2 to inhibit *S*. *pyogenes* SP-STKK.

### Inh2-B1 serves as “antibiotic resistance breaker” and rejuvenates the lost activity of cell wall acting antibiotics

**Figure 4:**
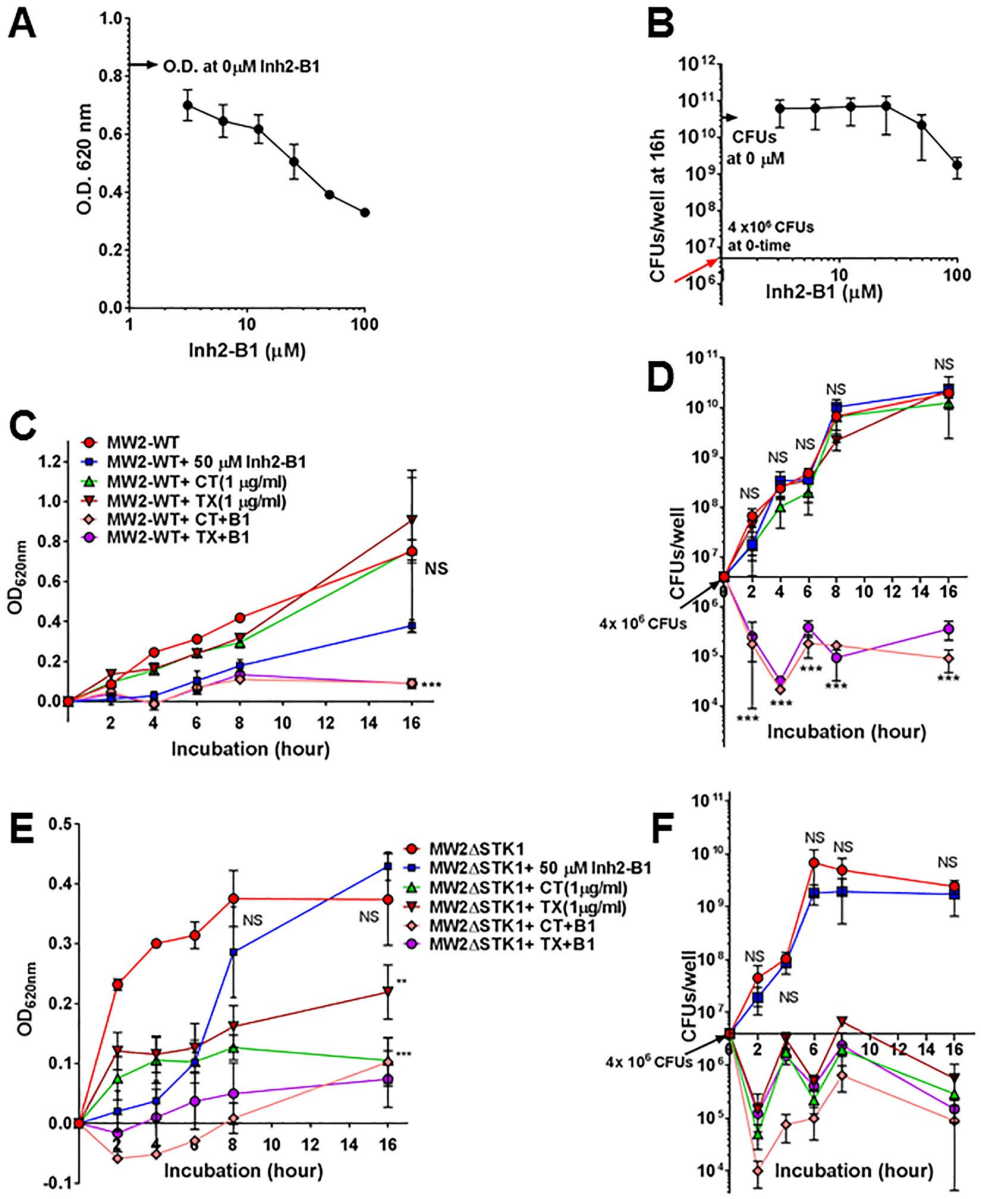
Effects of Inh2-B1 enhances the bactericidal activity of Ceftriaxone and Cefotaxime. Time-to-kill experiment shows Optical Density (OD) at 620 nm and viable bacterial counts (colony forming units) of (**A**,**B**) MW2-WT at different time intervals, when grown in the presence and absence of Inh2-B1 inhibitor. Optical Density and corresponding Colony forming units of (**C**,**D**) MW2-WT and (**E**,**F**) MW2ΔSTK1 cultures in the presence or absence 50 µM Inh2-B1, 1.51 µM (1 µg/ml) Ceftriaxone, 2.125 µM (1 µg/ml) Cefotaxime, and combination of Inh2-B1 plus Ceftriaxone, and Inh2-B1 plus Cefotaxime. The experiments were carried out as described in the Materials and Method section using Muller-Hinton broth and seeded with 4 × 10^6^ CFUs of *S*. *aureus* MW2-Wild type or MW2ΔSTK1 strains per well. Each data points represent Mean ± S.D. P values of significance (**<0.01, ***<0.001) were determined using the *t*-test with Welch’s corrections. NS-Not significant.

Since the STK1 is not essential for bacterial survival and its absence results in retardation of growth, we performed an end-point bactericidal assay employing the Checkerboard titration method followed by a time-to-kill assay using the broth dilution method. The 96-well plate-based Checkerboard titration assays were performed using the fixed number of CFUs of MW2 strain, and the survival of the bacteria was measured on solid agar plates as an end-point result. We noted the presence of ≥1CFU of the wild-type MW2 strain only in the wells that had ≤50 µM of Inh2-B1, ≤75.00 µM of Ceftriaxone or ≤110 µM of Cefotaxime. Further analysis revealed the growth of MW2 even up to a concentration of 90 µM Inh2-B1 (i.e., Minimum bactericidal concentration [MBC] of 100 µM), 125 µM Ceftriaxone (i.e. MBC 151.15 µM i.e., 100 µg/ml) or 176 µM of Cefotaxime (MBC of 220 µM i.e. 100 µg/ml). Additionally, in the presence of 50 µM of Inh2-B1, the MBC of Ceftriaxone for MW2 reduced to 0.59 µM, i.e., 0.39 µg/ml (256-fold reduction) (Fig. S7A), and that of Cefotaxime reduced to 27.5 µM, i.e., 12.5 µg/ml (8-fold reduction) (Fig. S7B). The cut-off value to designate clinical isolates of *S*. *aureus* for antibiotics, including Ceftriaxone (TX) and Cefotaxime (CT) as susceptible, is less than 4 µg/ml (i.e., <4.8 µM of TX, and <7 µM of CT). These results, thus, indicated that Inh2-B1 serves as an antibiotic resistance breaker and enhances the bactericidal activity of Ceftriaxone more efficiently than of Cefotaxime on drug-resistant *S*. *aureus* (Fig. S7). To substantiate this static culture/endpoint-based results, in subsequent experiments, we, determined CFU counts of MW2-WT grown in the presence of various concentrations of Inh2-B1 over a period of 16 h. Even at 100 µM of Inh2-B1, MW2 showed more than 3 log increase in growth, i.e. from the starting inoculum concentration of 4 × 10^6^ CFU to 1.8 ± 1.0 × 10^9^ CFU growing (Fig. 4A and B). We, therefore, treated the observed growth inhibition in comparison to the wild-type (~2 log reduction) at a very high concentration of Inh2-B1 as resistant or as minimal inhibition. Such inhibition could be an outcome of the non-specific toxic effect associated with the compound. Similarly, we also determined CFU counts of MW2-WT and MW2ΔSTK1 strains each grown in the absence or the presence of 50 µM of Inh2-B1 and 1.5 µM Ceftriaxone (1 µg/ml) or 3 µM Cefotaxime (1 µg/ml) for 16 h employing the broth dilution method. In the presence of only Inh2-B1 or only Ceftriaxone, or Cefotaxime, the growth pattern of the wild-type remained unaltered even after 16 h of incubation (Fig. 4C,D). As expected, in the absence of STK1 activity, the growth of MW2ΔSTK1 reduced significantly in the presence of Ceftriaxone because of the increased susceptibility to cell wall acting antibiotics (Fig. 4E and F). In the presence of Inh2-B1, the growth pattern of the MW2 wild-type was slightly retarded and mimicked to that of MW2ΔSTK1 as a result of the chemical inactivation of STK1 activity as described above (Fig. 4A and B). However, in the presence of the mixture of 1.5 µM Ceftriaxone (1 µg/ml) and 50 µM Inh2-B1, the growth of the MW2 wild-type was dramatically inhibited similarly to MW2ΔSTK1 (Fig. 4C and D). Further, to address whether MW2 or STK1 could become refractory to a potential combined therapy, we grew the *S*. *aureus* MW2 wild-type strain in the presence of suboptimal dose of Inh2-B1 alone and combination with Ceftriaxone and Cefotaxime (25 µM Inh2-B1 and 3 µM of Ceftriaxone or 3 µM Cefotaxime) for consecutive 10 generations. The passaged strain in both cases did not show any changes in the *stk1* gene as determined by PCR and DNA sequencing indicating that that STK1 activity remained intact. Further, the bactericidal activity of Ceftriaxone also remained intact in the presence of 50 µM Inh2-B1 as described above. Together these results supported the notion that STK1 is likely playing an important role in certain essential and physiologically relevant cellular activities, such as cell wall biosynthesis. These results confirmed that Inh2-B1 indeed serves as an antibiotic resistance breaker.

Inhibitory activity of Inh2-B1 on *S*. *aureus* MW2 at high concentration prompted us to investigate whether Inh2-B1 would also act synergistically with antibiotics other than those that target cell wall biosynthesis. We examined changes in antibiotic susceptibility patterns of MW2-WT for other antibiotics in the presence and absence of Inh2-B1 by E-test™. In the same assay, we used the MW2ΔSTK1 mutant as a positive parallel control to compare its susceptibility pattern with that of the MW2-WT strain in the presence of Inh2-B1. Our results clearly demonstrated that Inh2-B1 did not change susceptibility patterns of any of the 6 antibiotics (Ciprofloxacin[CI], Clindamycin [CM], Erythromycin [ER], Meropenem [MP], Ofloxacin [OF], and Tetracycline [TC]) for the MW2 strain. MIC values of all antibiotics for MW2-WT in the presence and absence of Inh2-B1 and those of MW2ΔSTK1 remained unaltered indicating that the observed synergistic activity of Inh2-B1 is mediated essentially via STK1 inhibition and by interfering with cell wall biosynthesis (Fig. S8).

### Inh2 and Inh2-B1 treatment affect the cell wall biosynthesis via regulating expression of cell wall hydrolase-encoding genes

**Figure 5:**
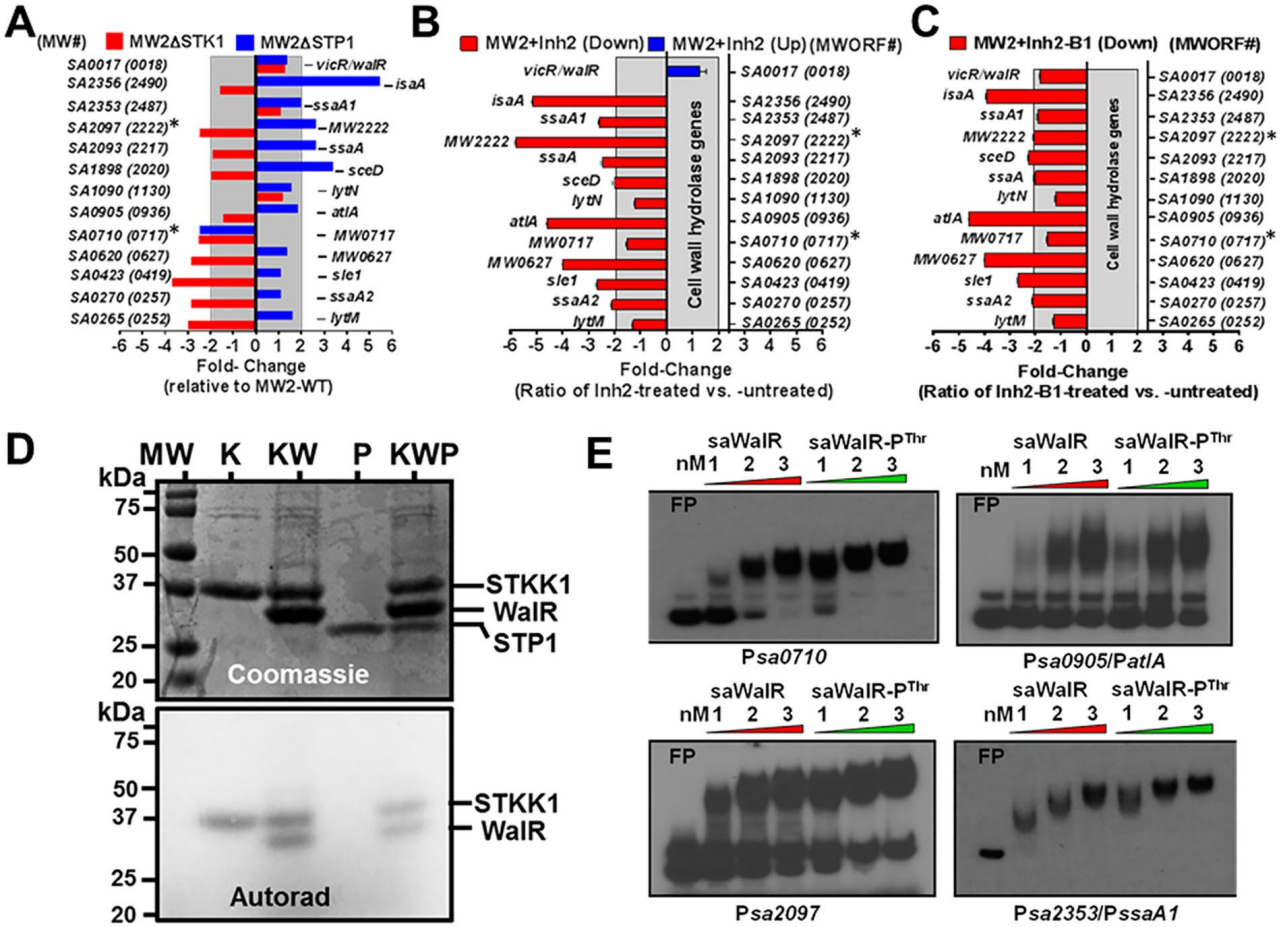
Inh-2 and Inh2-B1 treatment adversely affect the expression of genes encoding cell wall hydrolases in *S*. *aureus* via inhibiting STK1 activity. (**A**) qRT-PCR-based mRNA expression profiles of cell wall hydrolase genes in MW2ΔSTK1 and MW2ΔSTP1 mutants vs. MW2-WT show the reciprocal regulation in the absence of STK1 and STP1. Numbers shown on the Y-axis refer to ORF (open reading frame) numbers of the cell wall hydrolase-specific genes present in the *S*. *aureus* N315 genome and numbers within parentheses refer to the corresponding genes present in the MW2 genome. qRT-PCR-based mRNA expression profiles of cell wall hydrolase genes in *S*. *aureus* MW2-WT wild-type strain treated with (**B**) Inh2 and (**C**) Inh2-B1. Each horizontal bar represents an average value (Mean ± S.D.) from three different experiments each with three technical replicates. (**D**) STK1 and STP1-mediated reversible phosphorylation of *S*. *aureus* WalR. Phosphorylation assays were carried out in the phosphorylation buffer containing γ^32^P-ATP as described in the Materials and Methods section. The upper panel in (**D**) represents the Coomassie-stained SDS-page gel with differentially migrated *S*. *aureus* STKK1 (K), WalR (W), and STP1 (P) proteins. Each lane represents the combination of one or more of these proteins present in the phosphorylation, dephosphorylation, and control reaction mixtures. The lower panel is a corresponding autoradiogram of the upper panel. Numbers on the left represent molecular weight markers (MW) in kDa. (**E**) EMSAs showing the impact of the binding of different concentrations of the recombinant WalR and the STKK1-phosphorylated WalR to the promoter region of four genes-specific (SA0710, SA0905, SA2097, SA2353) γ^32^P-DNA probe on their electrophoretic migration in the gel.

In *S*. *pyogenes*, SP-STK and SP-STP reciprocally regulate the expression of the key cell wall hydrolase, CdhA, possibly via reversible phosphorylation of WalR^37, 42–44^ as also recently reported in *B*. *subtilis* ^45^. The WalR-mediated regulation of cell wall hydrolases is also observed for *S*. *pneumonia*e^46^ and *S*. *aureus* ^47^. Cell wall hydrolases play a crucial role in the modulation of the cell wall structure, muropeptide-turnover/recycling, cell shape, cell division, growth as well as innate immune detection and ultimately fitness, virulence, and drug resistance^48, 49^. A previous report on transcriptome analysis of *S*. *aureus* NCTC 8325^50^ mutants lacking respective *stk1* gene has shown that the observed defective cell wall is likely due to the altered regulation of cell wall hydrolases required for the cell wall biosynthesis. We, therefore, examined the expression profile of genes encoding cell wall hydrolases in *S*. *aureus* MW2 wild-type strain and mutants lacking STK1 and STP1 by qRT-PCR analysis. We then compared these results with those obtained with wild-type strain treated with and without Inh2 and Inh2-B1. This analysis revealed reciprocal regulation of 9 out of 13 tested genes in MW2ΔSTK1 and MW2ΔSTP1 mutants when compared to the MW2-WT strain (Fig. 5A). Out of these nine differentially altered genes, the MW2ΔSTK1 mutant strain displayed significantly reduced transcript abundance (i.e. exceeding cut off limit of 2-fold) of four (SA0620, SA0423, SA270, and SA0265) out of those nine differentially regulated genes. Similarly, the MW2ΔSTP1 mutant displayed significantly upregulated transcript abundance of three (SA1898, SA2093, and SA2356) of those nine genes. In contrast to these patterns, we observed unique differential regulation of two remaining genes (SA0710, and SA2097). While the transcript abundance of SA0710 was found to be significantly decreased (>2-fold) in MW2ΔSTK1 as well as MW2ΔSTP1 mutants, the reciprocal regulation of another gene, SA2097, was observed in the absence of *stk1*, as well as *stp1* genes i.e. decreased and transcript abundance in MW2ΔSTK1 and MW2ΔSTP1 mutants respectively. Notably, there was no significant change in the expression level of the *walR*/*VicR* (SA0017/MW0018) gene in the absence of either the *stk1* or *stp1* gene, indicating that the observed differential regulation of cell wall hydrolase genes was not directly due to the differential transcript abundance of the *walR* gene. Similar to the down-regulation of cell wall hydrolase genes in the MW2ΔSTK1 mutant, treatment of the MW2-WT strain with Inh2 and Inh2-B1 also caused the down-regulation of cell wall hydrolase-encoding genes (Fig. 5B and C). The two-component regulator, WalR, has been shown to modulate the transcription profile of several cell wall hydrolase genes, including those encoding LytM, AtlA, SsaA, and IsaA proteins in *S*. *aureus* ^50, 51^. Further, the deletion of either the *stk1* or *stp1* gene did not alter the *walR* transcript abundance. We, therefore, examined whether the observed altered transcription profile was due to the post-translational modification of WalR. To test this hypothesis, we subjected recombinant WalR to the STK1- and STP1-mediated *in vitro* phosphorylation and dephosphorylation. *In vitro* phosphorylation results, revealing STK1- and STP1-mediated reversible phosphorylation of the recombinant WalR protein concurred with our hypothesis and the notion that STK1 might modulate and fine tune the expression of cell wall hydrolases via altering the functional status of WalR (Fig. 5D). To confirm this, we examined whether posttranslationally modified WalR differentially binds to promoters of some of the cell wall hydrolase genes, which showed differential transcript abundance. To determine this binding, we employed an electrophoretic mobility shift assay (EMSA), using recombinant WalR and STKK1-phosphorylated WalR, and determined its ability to bind to the promoter regions of four genes. As described above (Fig. 5A), two of these genes (SA0710 and SA2097) were differentially regulated, and two showed no changes in their expression when either the *stk1* or *stp1* gene was deleted, or when STK1 activity was chemically inhibited with Inh2 or Inh2-B1 (Fig. 5B and C). EMSA was performed as described in the Materials and Methods section. The results showed that the STKK1-phosphorylated WalR protein, in comparison to the unphosphorylated WalR, bound specifically to the γ^32^P-labeled SA0710 and SA2097 DNA probe with significantly high efficiency (>2 fold Bound/free ratio) (Fig. 5E). However, this increased promoter binding efficiency of WalR-P was not observed for other two genes (SA0905 and SA2353), which served as negative controls (Fig. 5E). In the presence of 100× cold-probes specific to all four genes, the binding of corresponding γ^32^P-labeled DNA probes to WalR was completely inhibited (Data not shown). Together, these results revealed the impact of the Inh2-B1-mediated chemical inhibition of the STK1 activity similar to the deletion of the *stk1* gene in *S*. *aureus* indicating that STK1 contributes to the WalR binding to its certain promoters and is able to modulate the transcription of corresponding downstream genes. Thus, the observed effect of Inh2-B1 is likely mediated via inhibiting STK1-mediated WalR phosphorylation and modulating the ability of phosphorylated WalR to regulate transcription of cell wall hydrolase genes and resulting expression of cell wall hydrolases and subsequently the cell wall biosynthesis in *S*. *aureus*.

### Inh2-B1 inhibits *S*. *aureus* biofilm formation

**Figure 6:**
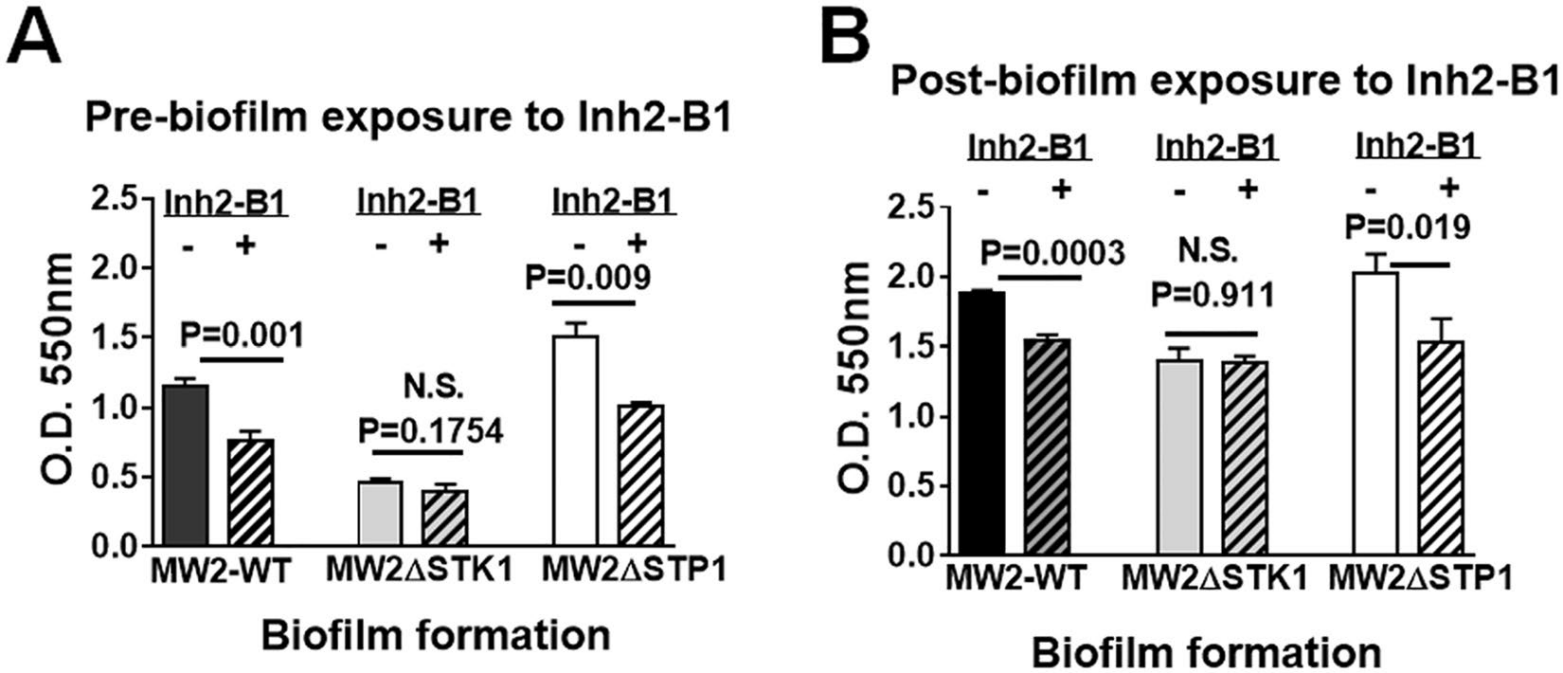
Inh2-B1 disrupts the formation *S*. *aureus* biofilms. (**A**) Biofilms formation by *S*. *aureus* MW2 wild-type, MW2ΔSTK1 and MW2ΔSTP1 mutant strains in the absence and presence of 25 µM Inh2-B1 after 48 h. (**B**) Effect of Inh2-B1 (25 µM) on the preformed biofilms by *S*. *aureus* MW2 wild-type, MW2ΔSTK1, and MW2ΔSTP1 mutant strains. Biofilm formation was quantitatively estimated after 48 h of incubation by the crystal violet staining method. Bar diagrams represent average values (Mean ± S.D) obtained for different parameters from three independent cultures each with three technical replicates. P < 0.05 was treated as a significant difference.

*S*. *aureus* biofilm formation is a complex multifactorial phenomenon^51, 52^. Since cell wall hydrolases play a crucial role in cell wall biosynthesis and the biofilm formation^51^, we sought to examine whether Inh2-B1alone can inhibit the biofilm formation by *S*. *aureus* employing the crystal violet stain method. The results shown in Fig. 6 revealed that the *S*. *aureus* mutant lacking STK1 formed poor biofilms when compared to that of the wild-type (P = 0.0005). MW2ΔSTP1 mutant, on the other hand, formed comparable and robust biofilms comparable to the wild-type strain (P = 0.1361). In the same assay, when the biofilm formation was allowed to occur in the presence of Inh2-B1, the MW2 wild-type (P = 0.001), as well as the MW2ΔSTP1 mutant strains (P = 0.009) but not the MW2ΔSTK1 strain (P = 0.175) showed the significantly decreased formation of biofilms (Fig. 6A). Subsequently, we tested whether Inh2-B1 could reduce the preformed biofilms. Indeed as expected, further incubation resulted in the increased recovery of the biofilms in all strains. Despite this increase, we observed a similar pattern of the significant reduction in biofilm formation when individual biofilms of the MW2 wild-type (P = 0.0003) and MW2ΔSTP1 (P = 0.001) were further treated with Inh2-B1 for 48 h. Inh2-B1 treatment, however, did not affect biofilms of MW2ΔSTK1 (P = 0.911) (Fig. 6B). Further, the decrease in biofilm formation by Inh2-B1-treated MW2-WT and MW2ΔSTP1 was comparable to that of MW2ΔSTK1 treated with or without Inh2-B1 indicating that the observed reduction is due the chemical inactivation of STK1 activity in these strains. Together these results indicated that Inh2-B1, in addition to its role as an antibiotic resistance breaker, played a significant role in the regulation of biofilm formation and in the disruption of preformed biofilms via inhibiting the STK1 activity.

### Inh2-B1 displays cell-dependent minimal to no cytotoxicity

**Figure 7:**
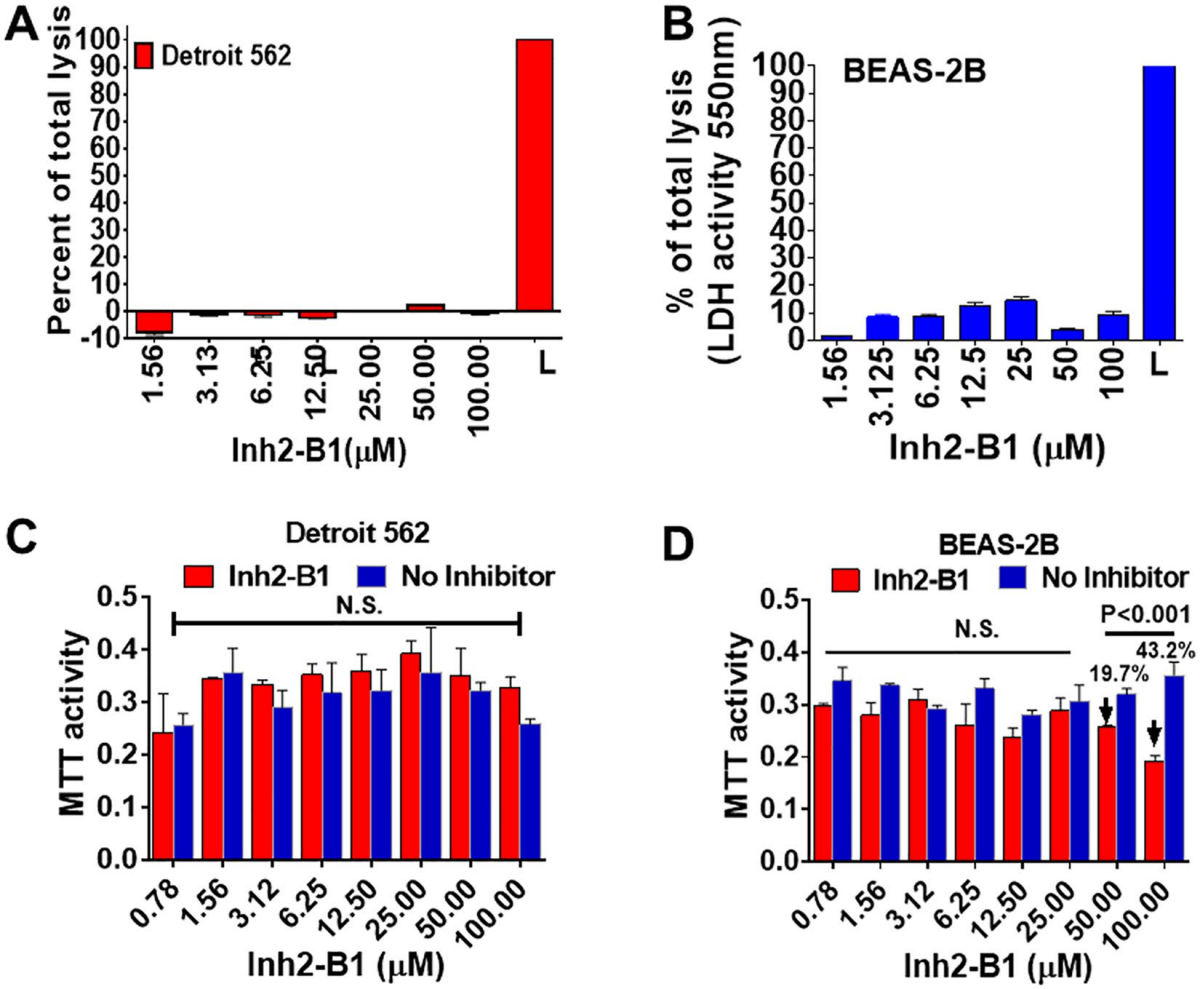
Human cell-dependent cytotoxic properties of Inh2-B1. (**A**,**B**) Lactate dehydrogenase (LDH) activity in Inh2-B1-treated human cell lines grown in 96-well tissue culture plates. LDH activity was measured in the supernatants of (**A**) Detroit 562, and (**B**) BEAS-2B cells lines treated with different concentrations of serially Inh2-B1 (serially 2-fold dilution- 100 µM–1.56 µM) for 24 h. LDH activity in individual wells was detected spectrophotometrically (λ = 490 nm) and normalized to the LDH activity of the whole cell lysates of confluent cultures of different cell lines (L), which was treated as 100% lysis. (**C**,**D**) Effect of different concentrations (100 µM- 0.78 µM) of Inh2-B1 on the proliferation of (**C**) Detroit 562, and (**D**) BEAS-2B cell lines grown in 96 well tissue culture plates as determined by the MTT test. The cells were treated with Inh2-B1 in a final volume of 100 µl for 24 h. At the end of incubation, tissue culture medium was removed and replaced with fresh 100 µl of the medium containing MTT solution (0.5 mg/ml) and further incubated for 4 h. The purple color of formazan salt crystals formed in each well was solubilized and spectrophotometrically analyzed (λ = 570 nm) as described in the Materials and Method section. Each bar shows the average (Mean ± S.D) reading of three different experiments each carried in triplicate wells. P < 0.05 treated as a significant difference. N.S. = not significant.

To investigate the potential therapeutic value of Inh2-B1, cytotoxic properties of the compound were first examined *in vitro*. Confluent cultures of an established human pharyngeal carcinoma cell lines (Detroit 562), and normal cell lines (BEAS-2B) were incubated in the presence of increasing concentrations of Inh2-B1 (1.56–100 µM), for 24 h in a CO_2_ incubator. Lactate dehydrogenase (LDH) activity was then assessed using a standard chromogenic substrate in the supernatant of each well. While BEAS-2B cell lines showed relatively higher cytotoxicity (~15%), Detroit 562 human lung cell lines showed less than 5% lysis (Fig. 7A and B). Since many kinase inhibitors have been therapeutically used to inhibit tumor cell proliferation but not lysis, we also examined the impact of Inh2-B1 on cell proliferation of these human cell lines by MTT assays. Inh2-B1 did not affect cell proliferation of Detroit 562 cell lines even at 100 µM concentration (Fig. 7C). However, we observed cell proliferation inhibition in BEAS-2B in the presence of Inh2-B1 only at 100 µM (~40%, P < 0.001) and 50 µM (~20%, P < 0.001) concentration (Fig. 7D). Together these results indicated that while some cell type may display toxic effects at a very high concentration of Inh2-B1, Inh2-B1 may remain nontoxic *in vivo* to most cells at a low therapeutic concentration.

### Inh2-B1 serves as an antibiotic-resistant-breaker and enhances bactericidal activity of Ceftriaxone and Cefotaxime in the *S*. *aureus* septicemia mouse model

**Figure 8:**
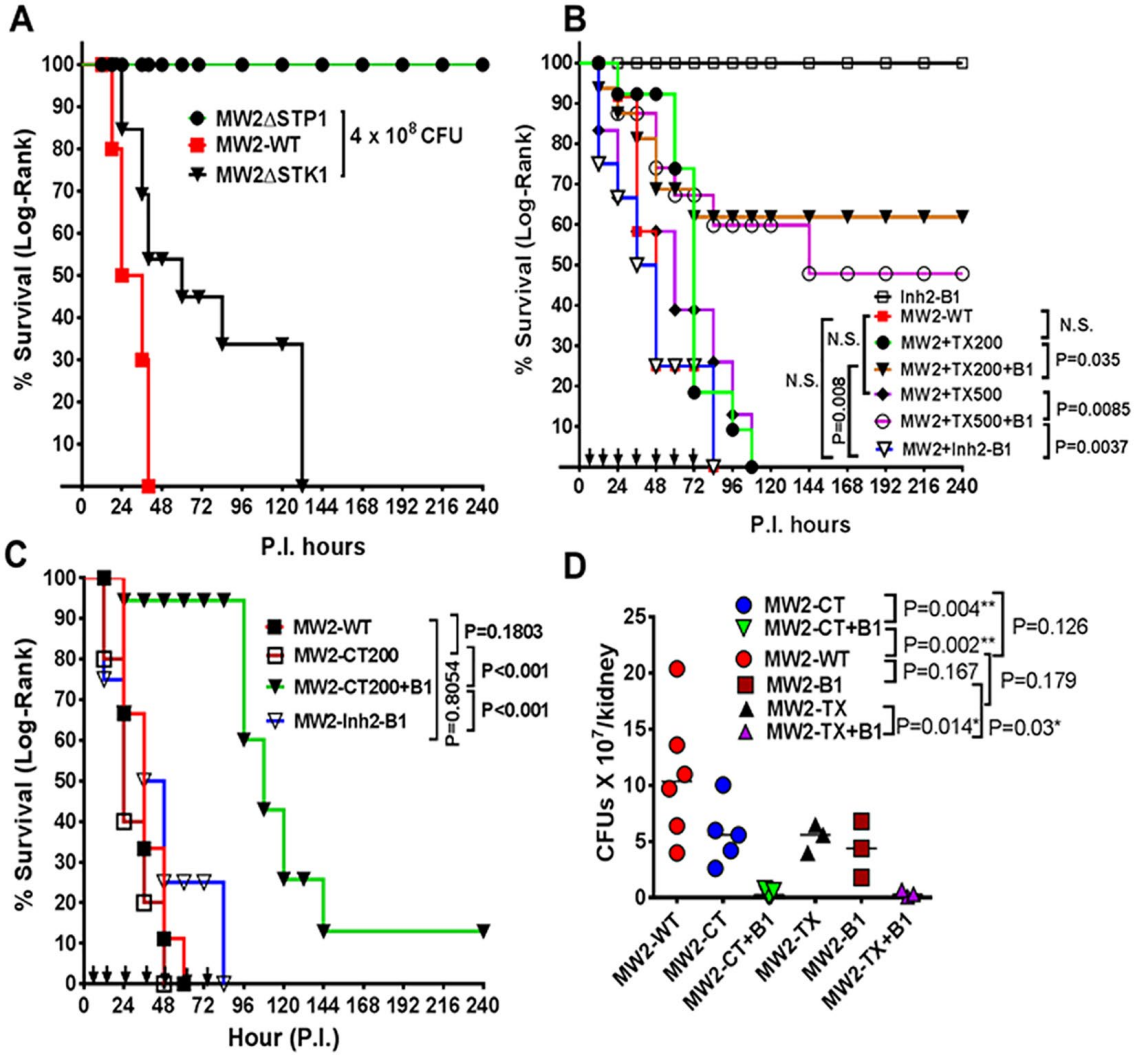
Inh2-B1 enhances the *in vivo* bactericidal activity of Ceftriaxone and Cefotaxime in the *S*. *aureus* mouse septicemia model. (**A**) Effect of the deletion of the genes encoding STK1 and STP1 on the virulence of *S*. *aureus* MW2 strain in the mouse septicemia model. A group of 10 CD-1 female mice (~20 g) were challenged retro-orbitally with *S*. *aureus* MW2-WT wild-type, MW2ΔSTK1 or MW2ΔSTP1 mutant strains (4.0 × 10^8^ CFU/100 µl) and were observed for ten days for mortality and morbidity. (**B**) Effect of treatment with only Inh2-B1 (5 mg/kg body weight) or Ceftriaxone (10 and 25 mg/kg body weight i.e. 200 and 500 µg/mouse), and combination of Inh2-B1 (5 mg/kg, 100 µg/mouse) and Ceftriaxone (10 and 25 mg/kg, 200 and 500 µg/mouse) on mice challenged with a lethal dose (4.0 × 10^8^ CFU/100 µl) of the MW2-WT strain. (**C**) Effect of treatment with only Cefotaxime (10 mg kg/body weight, 200 µg/mouse), and combination of Inh2-B1 (5 mg/kg body weight, 100 µg/mouse) and Cefotaxime (10 mg/kg body weight, 200 µg/mouse) on mice challenged with a lethal dose (4.0 × 10^8^ CFU/100 µl) of the MW2-WT strain. Mice were treated every 12 h for four days as described in detail in the text (arrows on the X-axis) and were observed for ten days for morbidity and mortality. P values less than 0.05 were treated as significant and were determined by the Log-Rank test using GraphPad Prism 6 software. N.S. not significant. (**D**) Determination of colony forming units in kidneys of treated and untreated challenged mice. To obtain relative differential bacterial loads in different treated and untreated challenged groups, 3–6 mice were sacrificed on Day 3 P.I The tissue extracts of kidneys were appropriately diluted, and CFUs were counted on tryptic soy agar (TSA). Results were statistically analyzed based on the median values obtained from each group (WT, CT, TX, B1, CT + B1 and TX + B1) using t-test with Welch correction. P value less than 0.05 was treated as significant. WT- wild-type, CT, –Cefotaxime, TX, Ceftriaxone, B1- Inh2-B1.

Mouse septicemia model was employed to evaluate STK1 as a therapeutic target and provide a proof of concept derived from the *in vitro* bactericidal assays described above that the STK1-inhibitor, Inh2-B1, can rejuvenate the bactericidal activity of Ceftriaxone *and* Cefotaxime *in vivo*. To determine the impact of the deletion of *stk1* and *stp1* genes on the pathogenicity of *S*. *aureus*, we challenged the CD-1 mice by inoculating bacterial suspensions of the corresponding mutant (MW2ΔSTK1 and MW2ΔSTP1), and the wild-type (MW2-WT) strains in the bloodstream of CD-1 mice via the periorbital venous plexus. Morbidity and mortality of the experimental animals were monitored twice daily for ten days. The untreated control mice challenged with the *S*. *aureus* MW2-WT strain died by 36 h post challenge (Fig. 8A). Mice that were challenged with the MW2ΔSTK1 mutant strain displayed a significantly delayed onset of acute infection when compared to infection with the wild-type MW2. However, all the challenged mice died by day 5 p.i. (Fig. 8A, MW2ΔSTK1 vs. MW2-WT, P = 0.0037). On the other hand, all mice challenged with MW2ΔSTP1 survived indicating that the deletion of *stp1* gene attenuates *S*. *aureus* virulence. Subsequently, we treated a group of ten CD1 mice intraperitoneally with compounds Inh2-B1 (5 mg/kg i.e. 100 µg/mouse), Ceftriaxone (10–25 mg/kg i.e. 200 and 500 µg/mouse), a combination of Inh2-B1 (5 mg/kg) and Ceftriaxone (10 and 25 mg/kg), or vehicle control at 12 h intervals. A total of nine doses were administered starting 4 h after the challenge with *S*. *aureus* MW2. The survival of challenged animals treated with therapeutic agents was recorded for ten days (Fig. 8B). The survival pattern of MW2-challenged mice that had received only Inh2-B1 or Ceftriaxone showed similar mortality patterns as was observed for mice that received no therapy (Inh2-B1 vs. untreated P < 1.0, Ceftriaxone vs. untreated P < 1.0). However, the MW2-challenged mice that received a combination therapy (Inh2-B1 5 mg/kg body weight, and Ceftriaxone 10 or 25 mg/kg body weight) showed significant (60%) survival (P < 0.003). Increasing the dose of Ceftriaxone from 200 to 500 µg/mouse did not provide proportionately increased protection. Further, mice that were treated with only Inh2-B1 or only the vehicle survived all throughout the observation period and showed no morbid signs of illness, supporting the minimal to nontoxic nature of Inh2-B1 observed by the cell-line based *in vitro* toxicity/proliferation assays (Fig. 7).

To determine whether the Cefotaxime was as effective *in vivo* as Ceftriaxone, we performed similar mouse protection studies as described above replacing Ceftriaxone with Cefotaxime using similar concentration (10 mg/kg body weight) in the presence and absence of Inh2-B1 (5 mg/kg body weight). The combination therapy (Inh2-B1 + CT) significantly prolonged the survival of mice (P < 0.001). The survival of the challenged mice in the absence of any therapy, or in the presence of only Cefotaxime remained the same, and all mice died within 36–48 hours unlike when the challenged mice were given a combined therapy (Fig. 8C). However, a number of survived animals at the end of 10 days were significantly low when the combined therapy included Cefotaxime instead of Ceftriaxone (Fig. 8B and C). Irrespective of this survival pattern, the presence of *S*. *aureus* MW2 burden on day 3 P.I. was significantly low (P = 0.002–0.014) in kidneys of mice, which received the combined therapy as compared to single therapy of Ceftriaxone, Cefotaxime, Inh2-B1, or no therapy (Fig. 8D). Together, these results demonstrated that inhibition of the kinase activity of STK1 enhanced the bactericidal activity of Ceftriaxone and Cefotaxime. Inh2-B1, thus, can serve as an “antibiotic resistance breaker” and hence, can be used in a combination therapy to repurpose the the so-called “failing” or “ineffective” cephalosporins against severe MRSA infection.

## Discussion

The development of organism-specific, and target-based (with a defined mechanism of action) therapeutic agent over broad-spectrum agents is thought to represent a good strategy to cope with the emergence of selective drug-resistant species^53, 54^. Some of these strategies include those that target virulence^55^, biofilm formation^56^, and antibiotic resistance^57^. Here, we have examined whether STK1 serves as a therapeutic target. It was clear from our previously published report that STK1, although plays a role in cell wall biosynthesis and the *S*. *aureus* mutant lacking STK1 becomes susceptible to the failing cell wall acting antibiotics and shows retarded growth^20^, it is not an essential protein such as WalR for *S*. *aureus* survival^47, 51^. Hence, the development of a therapeutic agent against STK1 can be envisaged only in breaking the MRSA resistance against and enhancing or repurposing the “failing/ineffective” cell wall acting antibiotics. Here, we have identified an STK1-inactivating compound, Inh2-B1, which can serve as an “antibiotic resistance breaker”. The quinazoline core structure of this compound has a precedent for many eukaryotic kinase inhibitors^31, 32^. Several amino-quinazoline-based inhibitors (Mitoxantrone, VI12112) have been described to inhibit the Ser/Thr kinase activity of mycobacterial PknB^33, 34^. However, this chemical inhibition did not result in growth inhibition even though the *pknB* gene is essential for growth^58^. In addition to the quinazoline-type inhibitor, tetrahydro benzothiophene compound, AX20017 has also been identified that inhibits the activity of PknG, a nonPASTA-containing mycobacterial kinase that enables bacterial survival within human macrophage^59^. This compound restores macrophage-mediated killing but does not inhibit the growth of extracellular bacteria. More recently, a sulfonamide-type STK1 inhibitor for *S*. *aureus* has also been identified that seems to enhance the *in vitro* activity of cell wall acting beta-lactam antibiotic, Nafcillin^60^. However, its efficacy in enhancing the bactericidal activity of Nafcillin *in vivo* against a lethal challenge of *S*. *aureus* is presently unknown. In the present investigation, we have shown that a derivative of Inh2, Inh2-B1, specifically inhibits the kinase activity of *S*. *aureus* STK1. Inh2-B1 shows minimal to none cell-dependent toxicity to human cells and enhances the bactericidal activity of the failing Ceftriaxone/Cefotaxime antibiotic against highly drug-resistant and pathogenic *S*. *aureus* both *in vitro* bactericidal assays and *in vivo* experimental septicemia mouse infection model.

The catalytic domain of the STKs of gram-positive pathogens is highly conserved^12^. Crystal structures of the kinase domain of mycobacteria STK (PDB ID: -1MRU)^61^ and that of *S*. *aureus* STK1 (PDB ID:4EQM)^38^ suggest a conserved mechanism of activation for these kinases. However, we were intrigued by the fact that Inh2-B1 did not inhibit the kinase activity of SP-STK of *S*. *pyogenes*. To understand this specificity, we employed structure-based docking analysis to determine molecular interactions of Inh2 and Inh2-B1 within the catalytic domain using the crystal structures of *S*. *aureus* STK1 (PDB ID- 4EQM)^38^. The amino acid sequence comparison of the kinase domain of Ser/Thr kinases of several Gram-positive pathogens^36, 38, 61^ has revealed a conserved ATP- binding site with a typical glycine-rich GRGG loop^38, 62^ (Fig. 3). The role of this loop is implicated in stabilizing the phosphates of ATP, and hence also called the P-loop^63^. Notably, the P-loop of *S*. *aureus* STK1 containing a unique tetra-glycine (G^17^GGG^20^) motif differs from those of STKs of other Gram-positive pathogens, which instead share the conserved G(R)GG motif (Fig. 3K). The inability of Inh2-B1 to inhibit the kinase activity of *S*. *pyogenes* SP-STK thus can be attributed to a different glycine-rich (GRGG) loop. Interestingly, Inh2-B1 but not ANP or Inh2 recognizes and interacts directly with the P-loop. Additional residues that are not recognized by inactivated ATP (ANP) or the lead molecule, Inh2, are L16 and G92. Additionally, among the six Inh2/Inh2-B1-interacting amino acid residues (S22, V24, A37, M87, T94, and F150; Fig. 3H and I), four of them except V24 and M87 are unique to the *S*. *aureus* STK1 (see the amino acid residues highlighted in pink in Fig. S9). This unique sequence, as well as the P-loop, constituted by L16, G17-20 residues present in *S*. *aureus* STK1, may allow Inh2-B1 to attain a pose for a stronger and a stable interaction with the catalytic domain of STK1. A recent study has shown that the species-specific PASTA domain and associated free peptidoglycans somehow determine the specificity of the STK enzyme for its activation in response to external stimuli^64^. Our molecular docking analysis of Inh2-derivatives showing the highest affinity (−11.6 kCal/mol) of Inh2-B1 for the kinase domain of STK1 (PDB 4EQM) suggests that the tertiary structure (catalytic pocket) formed by the glycine-rich loop and the surrounding networks of amino acids contribute to the species-specific kinase activity.

Gram-positive bacteria employ eukaryote-type-Ser/Thr kinases (STKs) and phosphatases (STPs) to regulate fundamental biological processes^12^. In *S*. *aureus*, STK1 controls bacterial virulence^14, 26, 27^ and plays a significant role in multidrug resistance traits^20, 22, 25^. Mutations in the *stp1* gene or the deletion of the *stp1* gene result in increased drug resistance with no effect on growth^20, 22, 25^. In this regard, the STP1 protein represents a poor candidate for a therapeutic target to control drug resistance problem despite the fact that *S*. *aureus* STP1 mutants are attenuated for virulence^25, 27^ as also observed for MW2 in the present investigation (Fig. 8A). The substrates of STKs, in general, include both stand-alone and TCS regulators^13^ including WalR as recently reported for *S*. *pyogenes* ^37^ and *B*. *subtilis* ^45^. Several important cell wall hydrolase genes, such as *lytM*, *atlA*, *ssaA*, have been reported to be down-regulated in *S*. *aureus* Stk1 mutant^50^ as has also been reported in conditional *walR* mutant of *S*. *aureus* ^51^. Here, we show that the expression of cell wall hydrolase genes is reduced in mutants lacking STK1 as well as in the Inh2-B1-treated MW2-WT. The reciprocally regulated expression of some of these genes in mutants lacking STP1 as compared to the isogenic mutant lacking STK1 derived from MW2 strain suggests that the *S*. *aureus* STK1 can also phosphorylate WalR *in vivo* as *in vitro* ^37, 45^. Our results showing reversible phosphorylation of *S*. *aureus* WalR by STK1 and STP1, and WalR binding to the promoter regions of certain genes support this notion and a possible mechanism of STK1–mediated regulation of staphylococcal cell wall hydrolases and mechanism underlying the “antibiotic-resistance-breaker” property of Inh2-B1.

The process of antibiotic resistance, biofilm formation, and cell wall biosynthesis are interrelated^51, 52, 56, 65^. The ability of Inh2-B1 to inhibit cell wall hydrolases as shown in the MW2ΔSTK1 mutant, and also the biofilm formation supports the previously published report wherein inhibition of the expression of WalR-dependent genes encoding cell wall hydrolases was found to be directly associated with the *S*. *aureus* ability to form biofilms^51^. The formation of biofilms results in decreased accessibility of antibiotics, which in turn promote the emergence of the drug-resistant population. The Inh2-mediated down-regulation of genes that encode cell wall hydrolases may relax peptidoglycan crosslinking^66^ and in turn, may facilitate penetration of other antibiotics or restore susceptibility to wall-acting antibiotics (Fig. 4). The fact that highly drug-resistant MW2ΔSTP1 mutant also became susceptible to Ceftriaxone and showed decreased biofilm formation suggests that the increased ability to form robust biofilms may serve as an additional factor that contributes to less penetration of effective antibiotics and increased drug resistance. In this regard, the present approach not only helps in enhancing bactericidal activity of the failing antibiotics but also serves as an anti-biofilm agent. Inh2-B1 in this context serves as a double-edged sword-“antibiotic resistant breaker”. One of the concerns that whether a potential combined therapy can fail if some mutation occurs in the STK1 encoding gene or indirectly through other hitherto unknown pathways. Our limited study with MW2 strain grown in the presence of a suboptimal dose of Inh2-B1 and Ceftriaxone/Cefotaxime did apparently show any changes in the STK1 activity. It is noteworthy that while mutations in the *stp1* gene of clinical isolates of *S*. *aureus* have frequently been observed but not in the conserved kinase domain of the *stk1* gene^14, 16, 22, 25, 29^. In fact, the *stk1* gene in all *S*. *aureus* genomes is completely conserved. Despite this negative finding, we do not eliminate a possible role of hitherto unknown factors^5, 55, 57^ that may indirectly contribute to decreasing the synergistic activity of Inh2-B1 on other cell wall acting antibiotics. Further investigation using multiple clinical isolates of *S*. *aureus* may provide useful insights into such the possibilities and in the development of an improved and more efficient combination therapy approach.

Mice infected with MW2ΔSTK1 mutant showed prolonged survival as compared to MW2-WT-infected mice as was reported before^26^. However, *S*. *aureus* strains are highly variable in their innate virulence properties and some highly drug-resistant strains such as *S*. *aureus* N315, are less virulent than CA-MRSA strains^67^. *S*. *aureus* Hla (α-hemolysin), an important virulence factor, is upregulated in ΔSTK1 mutant and down-regulated in ΔSTP1 mutant^27^. Non-hemolytic and completely attenuated nature of MW2ΔSTP1 could be, therefore, in part due to the down-regulated Hla-toxin (Fig. S1). However, we believe that the regulation of Hla-toxin is not directly influenced by STK as we did not observe changes in the hemolytic zone around the colonies of MW2-WT or other mutants in the presence of Inh2-B1. Hla has been shown to be regulated by multiple regulators both *in vitro* and *in vivo* ^68^. The therapeutic role of Inh2-B1 and the inhibition of STK1 activity are, therefore, not envisaged for decreasing the virulence (anti-virulence) although mice infected with MW2ΔSTK1 showed prolonged survival. MW2 wild-type infected mice, when treated with Ceftriaxone or Cefotaxime alone, were not protected, however, they were protected substantially by a combination therapy with Inh2-B1 and Ceftriaxone or Cefotaxime. The increased therapeutic concentration of Ceftriaxone 25 mg/kg) in a combination therapy did not offer better protection. The observed protection clearly indicated that the survival pattern is the reflection of the killing of the organism rather than delayed onset of the disease and prolonged survival. The reason of less efficient role of Inh2-B1 in increasing the bactericidal activity of Cefotaxime in comparison to Ceftriaxone is presently unknown. However, future experiments involving increasing the concentration of Cefotaxime and/or Inh2-B1, and testing the efficacy of other than the two third generation cephalosporins used in the present study against different Community –associated and drug-resistant pathogenic *S*. *aureus* strains may provide a better spectrum of this compound in terms of achieving increased *in vivo* efficacy.

In summary, to our best knowledge the present study provides the first comprehensive report on the identification STK1-specific and therapeutically relevant compound that can serve as an antibiotic resistant breaker and anti-biofilm agent. The compounds Inh2-B1 serves as an antibiotic resistance breaker by essentially inhibiting the kinase activity of *S*. *aureus* STK1 that contributes significantly to antibiotic drug resistance via modulating the function of cell wall biosynthesis machinery. Thus its action is effectively perceived in combinatioin therapy with certain cephalosporins that are deemed to be ineffective. Inh2-B1 poses minimal risks regarding cytotoxic or apoptotic activities on human cells. The latter property is essential since many kinase inhibitors have been successfully used based on their ability to cause apoptosis of human cells for tumor therapy. The inability of Inh2-B1 to cause no apparent toxic effects in experimental animals or minimal toxicity to normal or tumor cells, and ability to remain stable in the serum and body fluid, such as peritoneal fluid, are important initial safety features of this agent. The present findings thus clearly demonstrate the specificity of Inh2-B1 for *S*. *aureus* STK1 and support the future endeavors to develop an effective combination therapy with various ineffective cephalosporins and other cell wall acting antibiotics to repurpose them therapeutically and economically more effective against severe multidrug-resistant MRSA infections.

## Materials and Methods

### Bacterial strain, media, and cell lines

*S*. *aureus* strains MW2, a highly pathogenic and a multidrug-resistant strain was obtained from Network on Antimicrobial Resistance in *Staphylococcus aureus* (NARSA). *Streptococcus pyogenes* M1T15448 was obtained as described previously^37^. *Staphylococcus* strain was grown in tryptic soy broth (TSB) or TS agar. *Streptococcus* strain was grown in Todd-Hewitt broth supplemented with 0.5% yeast extract or 5% sheep-blood TS agar. *Escherichia coli* strain (DH5α, BL21 DE3-pLysS) were grown in Luria-Bertani (LB) or LB agar with or without ampicillin (100 µg/ml). All bacterial culture media were obtained from Difco (BD and Co, Sparks, MD). Antibiotics unless otherwise indicated were used at the following concentrations: 100 μg/ml of ampicillin and 10 μg/ml of chloramphenicol for *E*. *coli*, 10 μg/ml of erythromycin and 10 μg/ml of chloramphenicol and 1.5 μg/ml of anhydrotetracycline for *S*. *aureus*. Human pharyngeal carcinoma cell line (Detroit 562, CCL-138ATCC), and BEAS-2B (normal lung/bronchoalveolar immortalized cell lines, CRL-9609, ATCC), were grown in 24 well plates to obtain a confluent culture. Detroit 562 cell lines were grown in RPMI 1640-GlutaMax-I (GIBCO) tissue culture medium with 10% fetal bovine serum. BEAS-2B cell lines were grown in a serum-free medium, LHC-9, supplemented with epidermal growth factor and pituitary extract.

### Recombinant STKK1 and *in vitro* kinase assays

For the present study, we created recombinant His-tag-STKK1 along with part of the adjacent juxta-membrane domain (1- 280 aa) using the pET14b vector system and specific primers (Table-S1 and Table-S2) as described previously^20, 37^. The recombinant proteins were expressed in BL21 DE3 (pLysS) and purified using Ni^+2^-NTA-affinity chromatography, dialyzed against 10 mM Tris/HCl, pH 7.5, and stored as 10% glycerol stock (500 µg/ml) at −20 °C as described previously^20^. *In vitro* kinase assays were performed using 2 µg STKK1 in the presence of 1 µCi γ^32^P-ATP (specific activity 3000 Ci/mMol, PerkinElmer) at 30 °C for 45 min in a final volume of 30 µl phosphorylation buffer (50 mM Tris/HCl, pH7.5, 1 mM dithiothreitol, 5 mM MgCl_2_, and/or 5 mM MnCl_2_). The inhibition of autophosphorylation of STKK1 was carried out using this assay in the presence and absence of (50 µM) individual members of the 32 prescreened small molecule compound library. Phosphorylated proteins were separated by SDS-PAGE and subsequently identified by using Coomassie stain and autoradiography. The individual phosphorylated protein bands were then excised and subjected to radioactive counting using beta-scintillation counter for quantitative analysis. Experiments were repeated at least three times for statistical analysis. Compounds that showed more than 80% inhibition of the kinase activity was then further titrated (concentration range <1–150 µM) to determine its IC_50_ concentration for *S*. *aureus* STKK1 after preincubated for 5 min.

### Construction of Δ*stk1* and Δ*stp1* mutants

*S*. *aureus* MW2 mutants lacking *stk1* or *stp1* were created as described previously essentially using pMADΔ*stk1*chl and pKOR1Δ*stp1* vectors that were originally used to derive similar mutants from *S*. *aureus* N315 strain^20^ (Table-S1). Briefly, each plasmid construct was passed through *S*. *aureus* RN4220 strain before transformation into electrocompetent MW2. The Δ*stk1* mutant was selected on TSA containing chloramphenicol (10 µg/ml). The Δ*stp1* mutant was selected on TSA containing anhydrotetracycline followed by TSA and TSA with chloramphenicol. Chloramphenicol–sensitive transformants were confirmed on TSA plates with repeated passage to obtain the markerless Δ*stp1* mutant. The genetic integrity of mutants was confirmed by PCR and DNA sequencing using appropriate primers as described previously^20^. Growth curves of each mutant and the wild-type parent strain were measured in sterile 96-well microtiter plates using 1:250 diluted seed inoculum from the late log-phase culture. In addition to TSB, chemically defined medium (Teknova, Hollister, CA cat#4751) supplemented with 1% (w/v) glucose, maltose, lactose, or galactose was used to determine the impact on metabolic fitness. Growth curves were measured using PolarStar Galaxy spectrofluorimeter (BMG), which was preprogrammed to monitor changes in absorbance of the culture at 620 nm every 15  min at 37 °C for 16 h with horizontal shaking for 10 seconds before measuring the reading. The growth curve for each strain was then plotted using GraphPad Prism 6.

### Transmission electron microscopy (TEM)

TEM of the *S*. *aureus* strains was performed after fixing the late log-phase grown bacterial cells with 2.5% glutaraldehyde and 4% paraformaldehyde as described previously^20^.

### Quantitative real-time PCR

The late log phase-grown *S*. *aureus* strains MW2 wild-type, and isogenic ΔSTK1 and ΔSTP1 mutant strains, as well as *S*.*aureus* MW2 strain treated with 25 µM Inh2-B1 for 4 h, were subjected to lysostaphin treatment to obtain whole cell lysate as described previously^20^. Total RNA from the resulting lysate was first extracted by TRIZOL™ (Invitrogen/Thermo) and treated with RNase-free DNaseI. High-quality total RNA (RIN > 7.0, and 260/280 and 260/230 ratios >1.8) was then extracted by using RNA purification kit (Norgen, Canada) per manufacturer’s instructions and confirmed by an Agilent 2100 Bioanalyzer (Agilent Technologies, Palo Alto, CA). The first-strand cDNA from the total RNA was generated, and relative mRNA concentration was quantitated using SYBR Green qRT-PCR master mix and specific primers, using a Light Cycler® 480 real-time PCR machine (Table-S2). The results obtained for all samples (three biological replicates each in triplicates) were normalized to corresponding values of 16 S rRNA. All results including relative fold-changes in the mRNA expression (mutant vs. wild-type or treated vs. nontreated) for individual genes were analyzed using Exor4 software (Roche Applied Science) as described previously^37^. Changes in expression ratios by more than 2-fold (up- or down-regulation) were considered as significant.

### Electrophoretic mobility shift assays (EMSA)

EMSA experiments were performed using end-labeled ~250–300-bp (encompassing the promoter element upstream of four cell wall hydrolase-encoding genes SA0710, SA0905, SA2097, and SA2353. These probes were PCR-amplified using specific primers (Table-S2). Briefly, the binding reaction was performed in 35 μl reaction mixture containing labeled probe (20,000 cpm/ml) with purified 1–3 nM purified WalR in EMSA buffer (2 mg/ml poly dI-dC (Sigma), 10 mM Tris pH 7.5, 35 mM KCl, 1 mM EDTA pH 7.5, 1 mM DTT, 6% glycerol and 1 mM MgCl_2_). Phosphorylated and non-phosphorylated WalR were first incubated with poly dI-dC containing reaction buffer for 5 min at room temperature. The probe DNA was added, and the reaction mixture was incubated at 37 °C for 25 min. WalR-bound and free probe in the reaction mixtures were resolved by 4.5% non-denaturing polyacrylamide gel electrophoresis (200 V × 30 min) using 0.5 X TBE buffer and visualized by autoradiography. To establish the specificity of the STKK~P-phosphorylated WalR in binding to DNA, the autophosphorylated STKK~P was incubated with the ^32^P-labeled promoter probe as a control. Similarly, the 100-fold Cold-probe was mixed with the labeled probe in the EMSA to determine the specificity of WalR binding to its promoter. The band intensities were quantitated using AlphaInnotech ImageQuant densitometric software. The ratio of the concentration of nonphosphorylated WalR versus phosphorylated WalR required to achieve 50% of the maximum binding of phosphorylated WalR to different promoter probes was determined based on two separate experiments by GraphPad prism-6. These results were used to determine the relative binding efficiency of STKK-phosphorylated WalR to various promoters.

### Small molecule compounds

Previously, we were able to cherry pick 32 compounds that inhibited the growth of *S*. *aureus* RN4220 in the final concentration of ~40–60 µM range from the original small molecule library of 167,405 compounds^35^. This library was tested at the National Screening Laboratory for Regional Center of Excellence for Biodefense and Emerging Infectious Disease (NSRB) at the Harvard Medical School (Boston, MA). This prescreened NSRB library was the starting point of present investigation. Among these 32 compounds (serial number from Inh1 to Inh32), Inh-2 (N-(2,4-Dimethylphenyl)-5-oxo-1-thioxo-4,5-dihydro-[1,3] thiazolo [3,4-α] quinazoline-3-carboxamide) (initial screen hit 1391-A20) was identified as one of the three molecules that showed dose-dependent STK1 inhibition with lowest IC_50_ (San Diego, CA). Using this compound as the lead molecule, nine other compounds, each representing with modified side chain(s), were designed using the online ZINC software (http://zinc.docking.org)^69^ for the validation of biological properties of STK1 inhibition. These compounds were custom synthesized (mCule Co., SanDiego, California) (Fig. S4) at >95% purity and were initially stocked at 25 mM concentration in DMSO and stored at −80 °C. Subsequent dilution to the desired concentration was carried out in 50 mM Tris/HCl buffer in a pH range of 7.5–8.8. At this pH range, all molecules were found to be completely soluble yielding no visible pellet upon centrifugation (16,000 × g for 2 min).

### *In vivo* binding of the inhibitor to *S*. *aureus* STK1

An overnight culture of *S*. *aureus* MW2 strain was centrifuged, and the resulting bacterial pellet was resuspended in lysostaphin digestion buffer (50 mM Tris/HCl buffer, pH 7.5, containing 5 µg/ml DNAse/RNAse and protease inhibitor cocktail) in 1/10 the volume of the original culture volume. The latter was then subjected to lysostaphin treatment (15 U/ml). The lysostaphin digest was sonicated (5 sec pulse at every 10 sec for 10 min at 50% of 130 W, 20Kz amplitude), and the debris free whole cell lysate was obtained after centrifugation (10.000 × g, 10 min, 4 °C). The lysate was then equilibrated with binding buffer (10 mM Tris/HCl pH 8.0 and 150 mM NaCl) and passed through the solid phase- ATP-Separopore® 4B-CL column (BioWorld) pre-equilibrated with the binding buffer. The column was then washed with ten column volumes of binding buffer. Subsequently, the bound proteins were eluted with the 10-column volume of buffer containing 50 µM Inh-2 and Inh2-B1. *S*. *aureus* crude lysate, the last fraction of the column washing step, and eluted fractions were resolved by SDS-PAGE and electroblotted onto a PVDF membrane. The latter was probed with affinity purified rabbit anti-STK1 (IgG) (12) to determine the presence of STK1 eluted from the bound proteins from the crude lysate by the inhibitor in chemiluminescence-based Western Blot analysis. A similar experiment was performed using *S*. *pyogenes* whole cell lysates obtained from phage lysin-digested *S*. *pyogenes* M1T1 strain using the anti-SP-STK antibody as described previously using^36, 37^ to determine the specificity of the inhibitor for *S*. *aureus* STK1.

### *In Silico* determination of the binding of Inh2-derivatives to STK1 by molecular docking

To determine the interactive environment within the ATP-binding catalytic domain of STK1 for individual Inh2 derivatives, in silico molecular docking analysis was carried out employing Autodoc 4.0 software. To prepare the ligand, the program mol2chemfig (http://www.jcheminf.com/content/4/1/24), and a web server NCI/CADD (http://cactus.nci.nih.gov/translate) were used to generate the structure from SMILE. Furthermore, the structure of the compound was drawn with ACD Chem Sketch 11.0 (http://www.acdlabs.com/resources/freeware/chemsketch). Subsequently, its molecular geometry was optimized at the B3LYP/6-31 G (d, p) level up to a convergence in the energy of 10^−5^ AU using the Gaussian03 package. The crystal structure 4EQM (chain A) of *S*. *aureus* STK1 kinase domain was used for docking of the compounds after removing all the heteroatoms and crystal waters^38^.

The AutoDock 4.0 was used to conduct blind docking to identify the most likely binding site of the compound^70^. AutoGrid 4.0 was employed to build a 126 × 71 × 71 grid map covering the kinase domain with a spacing of 0.675 Å. The Lamarckian Genetic Algorithm was used for conformational sampling of the compound. For each docking simulation, 200 runs were carried out with 300 random individuals in the first population with 2.5 × 10^7^ energy evaluations and the 2.7 × 10^7^ number of generations. For the local search, the so-called pseudo-Solis and Wets algorithms were applied using the default parameter. From each docking run, the lowest-energy conformations of the compound were chosen for clustering with an RMSD cutoff of 2 Å. The conformations of the compound were then ranked and clustered based on their energy scores and populations.

Subsequently, for targeted or focused docking, grids with dimensions 50 × 50 × 50 and a spacing of 0.375 Å were focused on the biggest cluster that was obtained from the blind docking to get the most probable binding pose of the compound. Here, parameters of 2.5 × 10^6^ energy evaluations and the 2.7 × 10^6^ number of generations were applied. However, rest of the docking settings were kept similar to the blind docking step. The final conformations of the compound were ranked and clustered by energy and population scores. The VMD (Visual Molecular Dynamics) 1.9.1^71^ was used for analysis and image preparation.

### MIC and MBC determination

Growth inhibition of *S*. *aureus* strains (MW2-WT, MW2ΔSTK1 and/or MW2ΔSTP1) in the presence of Ceftriaxone (TX), Cefotaxime (CT), Ciprofloxacin (CI), Clindamycin (CM), Erythromycin (ER), Meropenem (MP), Ofloxacin (OF) and Tetracycline (TC) was initially determined using the E-Test^R^ method (AB BIODISK North America Inc., Piscataway, NJ) on Muller-Hinton agar plates as described previously in the absence or the presence of 50 µM Inh2-B1^20^. Subsequently, the susceptibility of *S*. *aureus* MW2 strain to Ceftriaxone, Cefotaxime, Inh2, and Inh2-B1 was confirmed by the CLSI recommended serial dilution method using Muller Hinton broth^71^.

Minimum bactericidal activity (MBC) was measured using Muller-Hinton agar. Changes in the MBC of Ceftriaxone/Cefotaxime in the presence of serially diluted Inh2-B1 was determined in two steps. Initial screening was carried out using the Checkerboard method using Muller-Hinton agar^72^ (Fig. S7). The MBC was identified by determining the lowest concentration of antibacterial agent that displayed a reduction in the viability of the initial bacterial inoculum by ≥99.9% (absence of any colony forming unit). Subsequent time to kill assays were carried out using 50 µM Inh2-B1 and Ceftriaxone, Cefotaxime (1 µg/ml) in a final volume of 250 µl using a sterile 96-well (U-bottom) microtiter plate over a period of 16 h at 37 °C under constant rotation (120 rpm). Optical density and corresponding CFU counts were measured at every two-hour interval up to 8 hours with a final overnight reading at the end of incubation period. CFU counts were measured using appropriate dilution of 10 µl of properly mixed samples at each time interval. All experiments were performed in three biological replicates and the statistical analysis of the results obtained at each time point was carried out by the non-parametric t-test with Welch’s correction using GraphPad Prism 6 software.

### Cytotoxicity

Various concentrations (1.56–100 µM) of the small molecule compounds Inh2-B1 were tested for their cytotoxicity by incubating them for 24 h in the cell lines mentioned above. The cytotoxicity of compounds was determined by measuring the activity of lactate dehydrogenase (LDH) released from the damaged cells during incubation. The LDH assay was performed in triplicate wells. At the end of incubation, the plates were lightly centrifuged, and the culture supernatants were examined for the presence of LDH activity using Cytotoxicity Detection Kit (Promega) *per* the manufacturer’s instructions. LDH activity was detected spectrophotometrically (Molecular device, λ = 490 nm) and normalized to the total LDH activity (100%) in cell lysate of the untreated cells. Background value obtained with untreated culture supernatant was subtracted before data evaluation.

### MTT-assays

Impact of Inh2-B1 on cell proliferation was determined by MTT (3-[4,5–dimethylthiazol-2-yl]-2,5 diphenyl tetrazolium bromide)-based assay using Roche Colorimetric proliferation assay kit. Cell lines mentioned above were grown in 96-well tissue culture plates in the CO_2_ incubator at 37 °C. The cells were treated with Inh2-B1as described above in a final volume of 100 µl for 24 h. At the end of incubation, tissue culture medium from each well was removed and replaced with fresh 100 µl of the medium containing MTT solution (0.5 mg/ml) and further incubated for 4 h. The purple color of formazan salt crystals formed in each well was solubilized with the addition of 100 µl of 10% SDS solution in 0.01 M HCl and further incubating for 10–12 h. The solubilized formazan product in each well was spectrophotometrically analyzed using a microtiter plate reader (Molecular Devices, λ = 570 nm). Absorbance values of wells containing MTT reagents without cells and the untreated cultured cells with MTT reagents were considered as the background and negative controls. All tests were performed in triplicate wells. The average background value obtained from 10 wells was subtracted from the test values, and the corrected values obtained from untreated and treated cells were statistically analyzed by Student’s test. P < 0.05 was treated as a significant difference.

### Biofilm formation

The ability to form biofilms by *S*. *aureus* MW2 strain in the absence and presence of Inh2-B1 was determined using the crystal violet stain method as described previously^51^. Briefly, the assay was carried out in 24-well tissue culture plates in a final volume of 500 µl of TSB broth with or without Inh2-B1 (25 µM). The culture plates were then seeded with 1:1000 dilution of the freshly grown *S*. *aureus* cultures to late log phase (O.D_600_ = 0.8) and further incubated for 48 h. At the end of incubation, the culture medium from each well was carefully removed and stained with 1% Crystal violet for 5 min. The unbound stain was then removed by repeated washings with distilled water and air dried. The stained biofilms were then extracted and dissolved in 500 µl 100% of isopropanol. Aliquots of fully suspended biofilms (100 µl/well) in isopropanol from each well were spectrophotometrically (λ = 550 nm) analyzed using a microtiter plate reader (Molecular Devices). The test samples were analyzed after subtracting the background values obtained from wells containing an only medium. All results were obtained with three independent cultures each in three wells. In another set of experiments, Inh2-B1 (25 µM) was added to the 48 h-grown established biofilms of S. a*ureus* MW2 to determine the impact of Inh2-B1 on the preformed biofilms and its ability to disrupt or further formation of biofilms. For this, old medium was carefully removed to allow the minimal loss of the loosely attached apical part of the preformed biofilms and replace with 2 ml of fresh TSB medium with or without 25 µM Inh2-B1 inhibitor and incubated further for 48 h at 37 °C. Subsequently, the biofilm formation or its disruption was quantitatively estimated as described above and statistically evaluated.

### Ethical statement

Animal protection study describe in the present manuscript was carried out in strict accordance with the recommendations in the Guide for the Care and Use of Laboratory Animals of the National Institutes of Health and the NC3R^S^ recommended ARRIVE (Animal Research: Reporting of *In Vivo* Experiments) guidelines (https://www.nc3rs.org.uk/arrive-guidelines). All animal experiments, anesthesia procedures, and early removal criteria were observed and performed per the protocol (#2007A0134-R3) approved by the Ohio State University Institutional Animal Care and Use Committee (IACUC).

### Animal experiments

The virulence potential of the wild-type *S*. *aureus* MW2 wild-type and corresponding MW2ΔSTK1 and MW2ΔSTP1 mutants was assessed using the mouse-septicemia model. CD-1 mice (5 weeks old, 20–22 g, Charles River Laboratories, ten female mice/group and housed in a group of 5 mice/cage) were lightly anesthetized with isoflurane and injected retro-orbitally with the *S*. *aureus* MW2 or isogenic MW2Δ*stk1* mutant (4 × 10^8^ CFU/0.1 ml of PBS). Infected, as well as sham-infected animals, were observed for 10 days for survival/mortality and morbidity. For therapeutic studies, the animals were first challenged with MW2-WT strain as described above and subsequently administered with antibiotic and/or small molecule compounds. The latter was started 4 h after the challenge and subsequently 12 h apart for 4 days post infection. Thus, Inh2-B1 (5 mg/kg body weight in 0.3 ml vehicle buffer), Ceftriaxone (10 and 25 mg/kg body weight in 0.3 ml vehicle buffer), Cefotaxime (10 mg/kg body weight), combination of Ceftriaxone (10 mg and 25 mg/kg body weight in 0.3 ml vehicle buffer) or Cefotaxime (10 mg/kg body weight) with Inh2-B1 (5 mg/kg body weight in 0.3 ml vehicle buffer), or 0.3 ml of vehicle-buffer (50 mM Tris/HCl, pH 8.8, 1.67% [v/v] DMSO). Early removal and euthanasia criteria were applied to all experimental mice showing penultimate sign(s) of the unrecoverable stage of illness, including ruffled skin with a hunch back, loss of more than 20% of body mass as compared to unchallenged mice receiving mock treatment, dyspnea, and labored gasping. All experiments were performed twice. Data were combined, and percentage survival in each group was statistically evaluated by the Log-Rank test (Kaplan-Meier) using the GraphPad Prism 6 software. P < 0.05 was treated as a significant difference. On Day 3 P.I., 3–6 mice from different groups receiving treatment with a single antibiotic or combination therapy were euthanized and sacrificed, and kidney tissues were homogenized for determining the bacterial load, which was determined by counting the colony forming units in the homogenized tissue in sterile PBS buffer. Statistical analysis of these results was performed based on the median CFU counts and by employing the non-parametric t-test with Welch’s correction.

## Electronic supplementary material

Supplementary Information 
