# Supplementary material for: A novel STK1-targeted small-molecule as an “antibiotic resistance breaker” against multidrug-resistant Staphylococcus aureus
Source: Sci Rep. 2017 Jul 11;7:5067. doi: 10.1038/s41598-017-05314-z (PMC5505960; doi:10.1038/s41598-017-05314-z)
Supplement: Supplementary file 1 — Supplementary Information [file 41598_2017_5314_MOESM1_ESM.pdf]

Supplementary Information

A novel STK1-targeted small-molecule as an "antibiotic resistance breaker" against multidrug-resistant *Staphylococcus aureus*

Sashi Kant[1], Shailendra Asthana[2], Dominique Missiakas[3], Vijay Pancholi[1¶]

[1]Department of Pathology, The Ohio State University College of Medicine, Columbus, Ohio, USA, [2]Drug Discovery Research Center, Translational Health Science and Technology Institute, Faridabad-Gurgaon Expressway, Haryana, India; [3]Department of Microbiology, University of Chicago, Chicago, Illinois, USA.

Running title: STK1-specific inhibitor to prevent *MRSA* diseases *(49 characters including space)*

¶ Corresponding Author:
Vijay Pancholi
Department of Pathology
The Ohio State University
420 W 12th Avenue, TMRF-288
Columbus, OH-43210

Email: vijay.pancholi@osumc.edu

Contents:

Table-S1
Table-S2
Figure S1
Figure S2
Figure S3
Figure S4
Figure S5
Figure-S6
Figure-S7
Figure-S8
Figure-S9

Table-S1: Strains and plasmids used in the present study

S. No.	Plasmids	Purpose of the study	Reference
1	pET14b	*E. coli* expression vector (*amp*^{R}) with N-terminal histidine tag	Novagen
2	pET14B-STP1	pET14B plasmid with entire *SA1062* gene inserted between NdeI and BamHI	1
3	pET14B-STKK1	pET14B plasmid with kinase domain of SA1063 gene inserted between Nde and BamH1	This study
4	pET14B-WalR	pET14B plasmid with kinase domain of SA0017 gene inserted between XhoI and BamH1	This study
5	pMADΔSTK1chl	pMAD containing up- and downstream regions of SA1063 (*stk1*)flanking chl	1,2
6	pKOR1ΔSTP1	pKOR1 containing up- and downstream regions of SA1062(*stp1*)	1,3
7	PET14B-SP-STKK	pET14B plasmid with kinase domain of the SP-STP (SPy_1625, SP-STKK) encoding gene of *Streptococcus pyogenes* M1SF370 inserted between NdeI and BamH1 followed by HpaI and BAmH1 digestion and blunt end religation	4
**S. No.**	**Strains**	**Purpose of the study**	**Reference/Source**
1	DH5α	*E. coli* : *F– Φ80lacZΔM15 Δ(lacZYA-argF) U169 recA1endA1 hsdR17(rK–, mK+) phoA supE44 λ– thi-1 gyrA96 relA1:* used for cloning	Invitrogen
2	BL21(DE3)pLysS	*E. coli* : F^- *ompT hsdS*$_B$ (r_B^- m_B^-) *gal dcm(DE3)pLysS* (*Cam*^{r}) for heterologous protein expression	Invitrogen
3	RN4220	Restriction-deficient derivative of NCTC 8325-4	NARSA
4	*S. aureus* MW2 (BK9897)	Community-associated Methicillin-resistant *S. aureus* parent strain	NARSA
5	MW2ΔSTP1	MW2 lacking SA1062	This study
6	MW2ΔSTK1	MW2 lacking SA1063	This study
7	*Streptococcus pyogenes* M1T1 5448	For screening Inh-2-screening small molecule inhibitors under study, In vivo binding of SP-STK	4

References for Table-S3.

1. Beltramini AM, Mukhopadhyay CD, Pancholi V. "Modulation of cell wall structure and antimicrobial susceptibility by a Staphylococcus aureus eukaryotic-like serine/threonine kinase and phosphatase." Infect Immun. Vol. 77, no. 4. (April 2009.): 1406-1416.
2. Arnaud, M., A. Chastanet, and M. Debarbouille. 2004. New vector for efficient allelic replacement in naturally nontransformable, low-GC-content, gram-positive bacteria. Appl. Environ. Microbiol. 70:6887–6891.
3. Bae, T., and O. Schneewind. 2006. Allelic replacement in Staphylococcus aureus with inducible counter-selection. Plasmid 55:58–63.
4. Jin, H. and Pancholi, V. 2006. Identification and biochemical characterization of a eukaryotic-type serine/threonine kinase and its cognate phosphatase in Streptococcus pyogenes:Their biological functions and substrate identification. J. Mol. Biol. 357:1351-1372.

Table S2: Primer sequences used for construction of recombinant proteins, qRT-PCR analysis and probe amplification for EMSA. The open reading frame (ORF) numbers are based on the genome sequence data of *S. aureus* N315 strain.

**Primers used for the construction of recombinant proteins**			
**ORF**	**Name**	**Forward (5′-3′)**	**Reverse (5′-3′)**
SA1063	STKK	CTACGGCCATATGATAGGTAAAATAATAAATGAACG	CGCGGGATCCTTAGACATCTTCATTCGC
SA1062	STP	CTACGGCCATATGCTAGAGGCACAATTTTTTACTGATACTGGAC	CCGCGGATCCTCATACTTTATCACCTTCAATAGCCG
SA0017	WalR	AAACTCGAGATGCAAATGGCTAGAAAAGTT	TTTGGATCCCTACTCATGTTGTTGGAGGAA

**Primer for Real Time PCR**			
**ORF**	**Gene**	**Forward (5′-3′)**	**Reverse (5′-3′)**
SA0017	*walR/vicR*	TGGAAGTATGTCGTGAAG	GTGCTGGTTGTGAGTAAT
SA0265	*lytM*	TAGGTCCAGACGCGAGCTAT	CGTCTTTCGCATGACCACTA
SA0270	*ssa-like*	TCGGTATTGCTGGTGTCAAA	CAACACGCCAAACAACAATC
SA0423	*sle1*	CGAACTCAGGATCTGCAACA	CGCTGCGTTATCCCAGTTAT
SA0620	*ssaA homologue*	CCACCTGATCCACCATTAGG	GCTGGTTCAGCATCATCTCA
SA0710	*lytE*	CACAACAACATGGCACACAA	TGAAATCACGTCACCAGGAA
SA0905	*atlA*	AATGGTTGCATTAACGCTTGT	TTGCTGTTTTTGGTTGGACA
SA1090	*lytN*	TGGGTATGTCGAACAAAGCA	TTTCCGTTTGAAATTGCTGA
SA1898	*sceD*	AGGAAATGCAGGTCACGAAG	TTAGTTGCAGGTGCTTGTGC
SA2093	*ssaA*	AATGGCCGTTCAATCTCAAG	ACGTATGCAACGTGACCGTA
SA2097	*ssaA*	AGGTCAAGCACATCATGCAG	ACCGATTTCTCCGCCTACTT
SA2353	*ssaA1*	TAACCACACCAGCACCATGT	TGTTGGTGGGAAAATTGGTT
SA2356	*isaA*	CTGCAGGTGCTACTGGTTCA	ACAGCTGCGTTGATTTGTTG
SArRNA01	*16S*	GTTATCCGGAATTATTGGGCG	CCGGGCTTTCACATCAGACT

**Primers for probe amplification for EMSA**		
**Name**	**Forward (5′-3′)**	**Reverse (5′-3′)**
PSA0710	AGTACAATTCGGTAGATAGAGTTAG	CTTGCTGTCATTCCTTTGCTGTTAG
PSA0905	AGTTGTATCTATTTTAGAAACATTTGT	TTCTATTTATTACTCCTAACAT
PSA2097	TCCTATTAATTATCTGTTAATCTC	GAATAAAGTCCTCCAAAGTTCTAT
PSA2353	ATCCTCCCAATAATCAAAACACTCT	CTTGATGCACTAAACTTTTGAAATAT

Restriction sites are in underlined

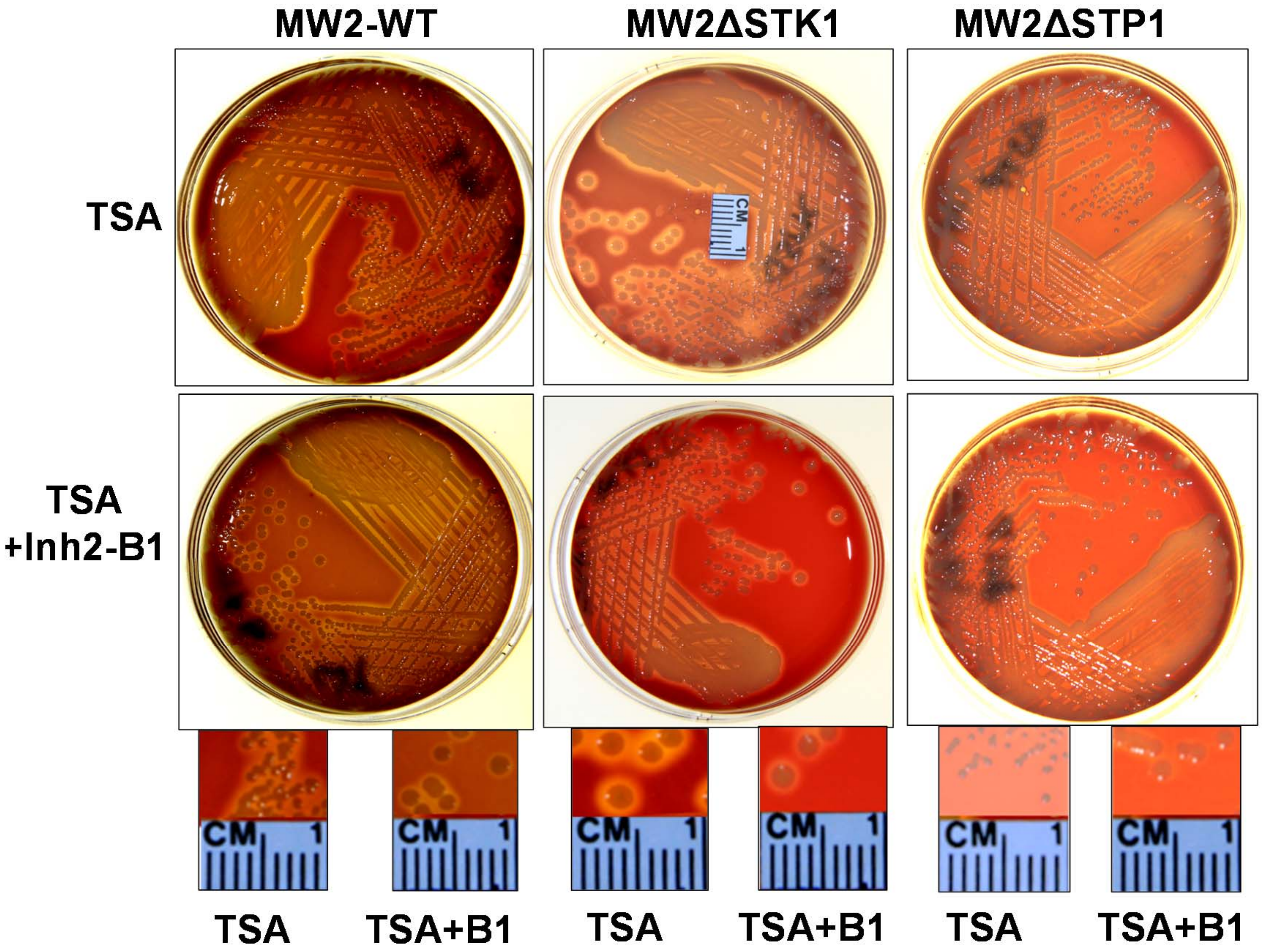

Fig. S1. Colony morphology and hemolysis patterns of *S. aureus* MW2-WT wild-type, and MW2ΔSTK1 and MW2ΔSTP1 mutant strains on TSA blood agar plates incorporated with or without 50μM Inh2-B1. See the increased hemolysis around the colonies of the MW2ΔSTK1 mutant and absence of hemolysis around those of the MW2ΔSTP1 mutant as depicted in the enlarged portion of certain representative colonies shown under each panel of the strain. One centimeter bar is include to facilitate the determination of the relative size of the colonies and corresponding hemolytic zones around them.

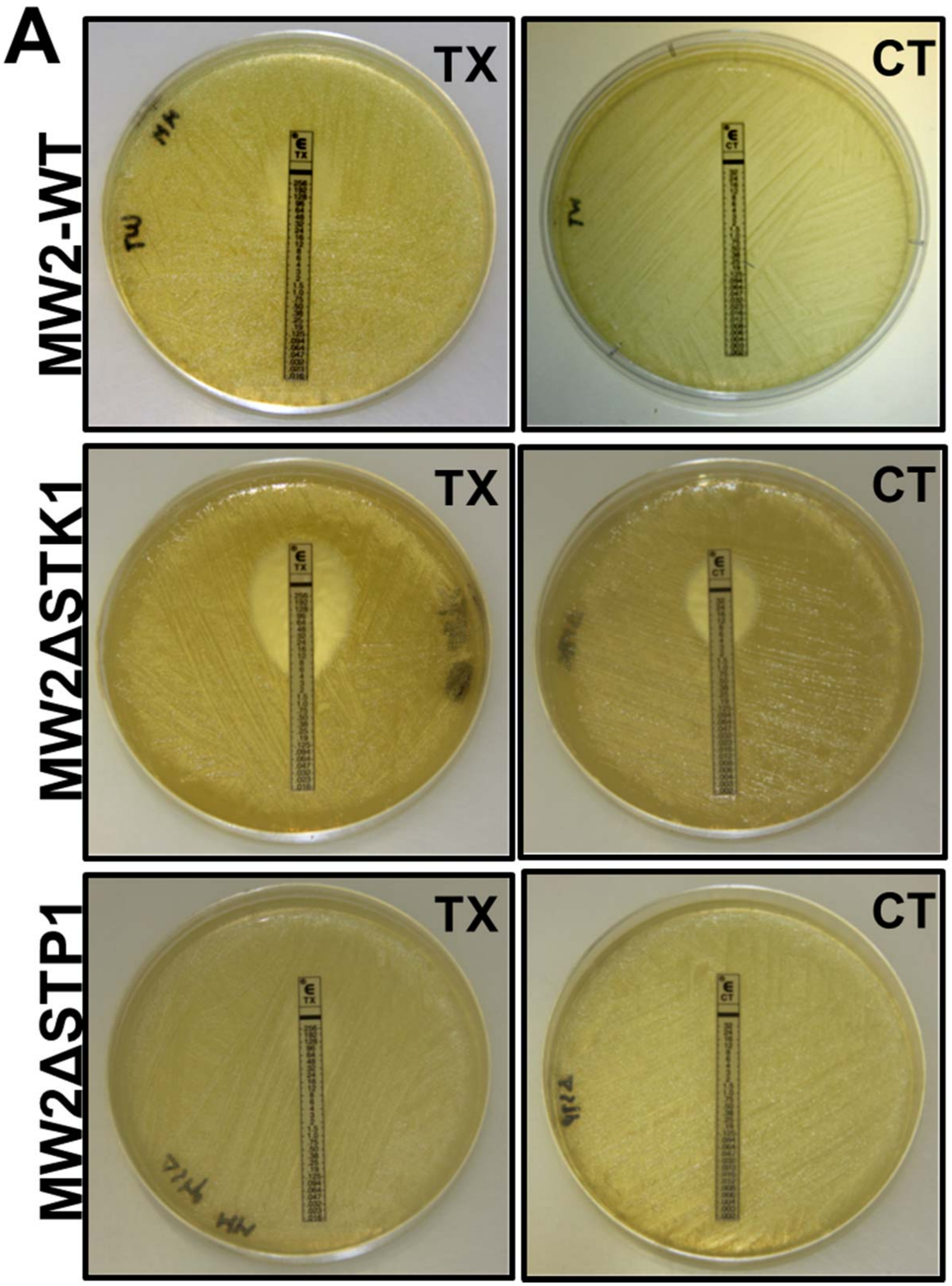

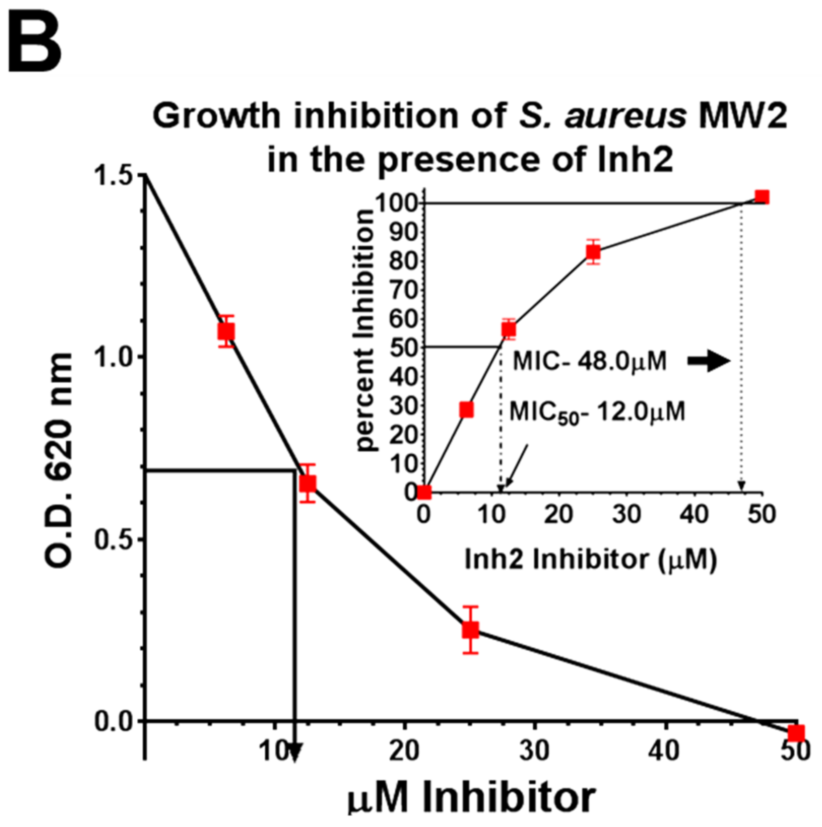

Fig. S2 Determination of susceptibility of *S. aureus* MW2 (WT, ΔSTK1 and ΔSTP1) strains to Ceftriaxone, Cefotaxime and Inh2 using Muller Hinton-II media. (A) E™-test strip-based MIC determination. Cefotriaxone (TX) and Cefotaxime (CT) using MH-II agar plates. (B) Susceptibility of *S. aureus* MW2-WT to Inh2 as determined by the broth dilution method using Muller-Hinton-II broth containing different concentrations of Inh2.

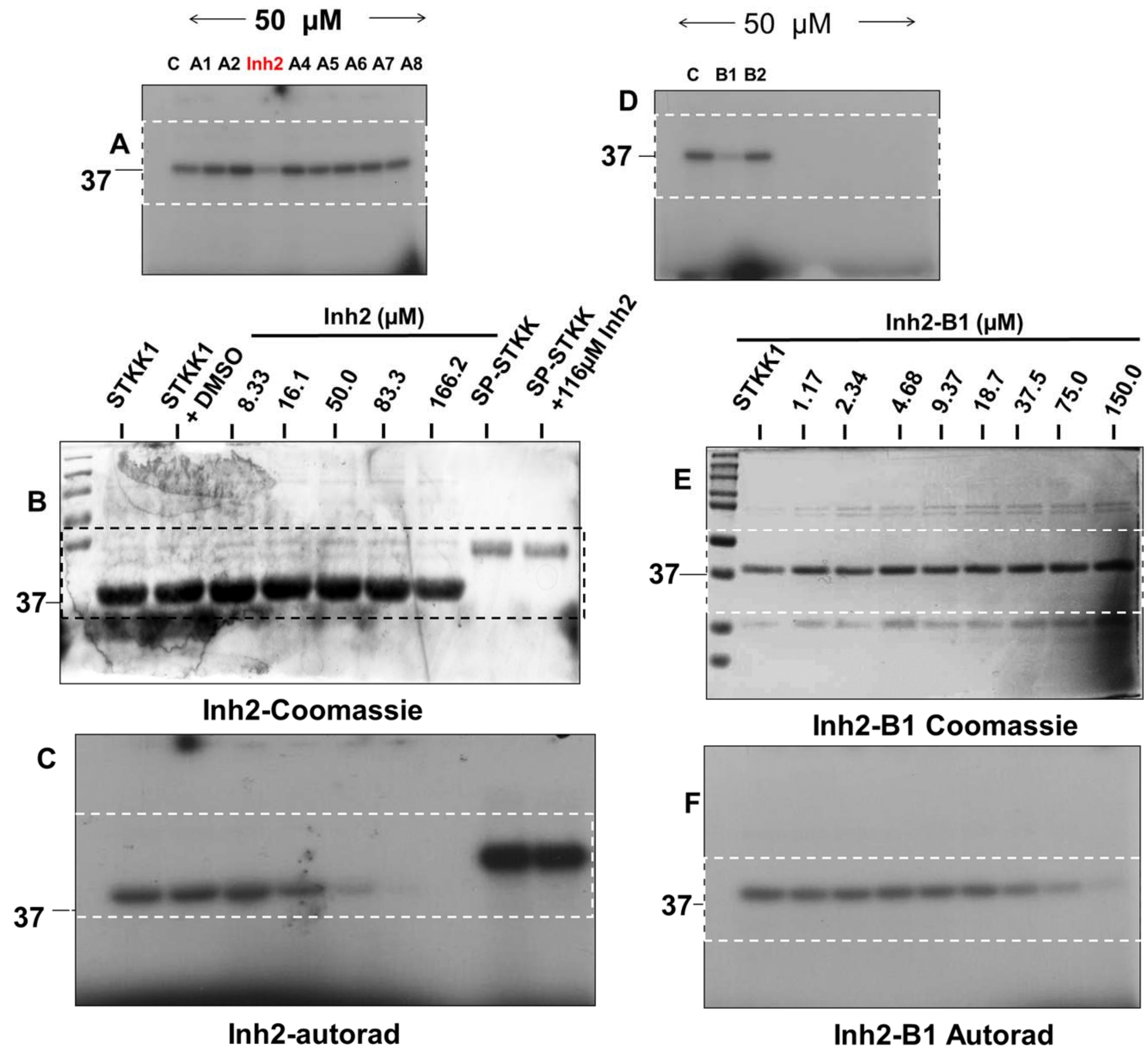

Fig S3. Screening of Inh2-derivatives for STKK1 phosphorylation inhibition. (A and D). Screening of Inh2-derivatives (50 μM as indicated) for their ability to inhibit the kinase activity of *S. aureus* STKK1 . (B and E) Coomassie-stained gels and corresponding autoradiograph (C and F) show the dose-dependent inhibitory activity of a small molecule inhibitors, (B) Inh2 (N-(2,4-Dimethylphenyl)-5-oxo-1-thioxo-4,5-dihydro [1,3] thiazolo[3,4-α] quinazoline-3-carboxamide) and (E) Inh2-B1 [Methyl 5-oxo-3-(phenyl carbamoyl)-1-thioxo-4,5dihydro[1,3]thiazolo[3,4-a]quinazoline-8-carboxylate] targeted to *S. aureus* STKK1 (the soluble kinase domain of STK1, 280 aa). The dotted rectangles represent the portion of each picture shown in Fig 2 in the main text.

A

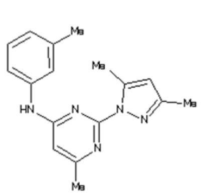

N-(3,5-dimethylphenyl)-2(3,5-dimethylpyrazol-1-yl)-6-methyl pyrimidine -4 amine

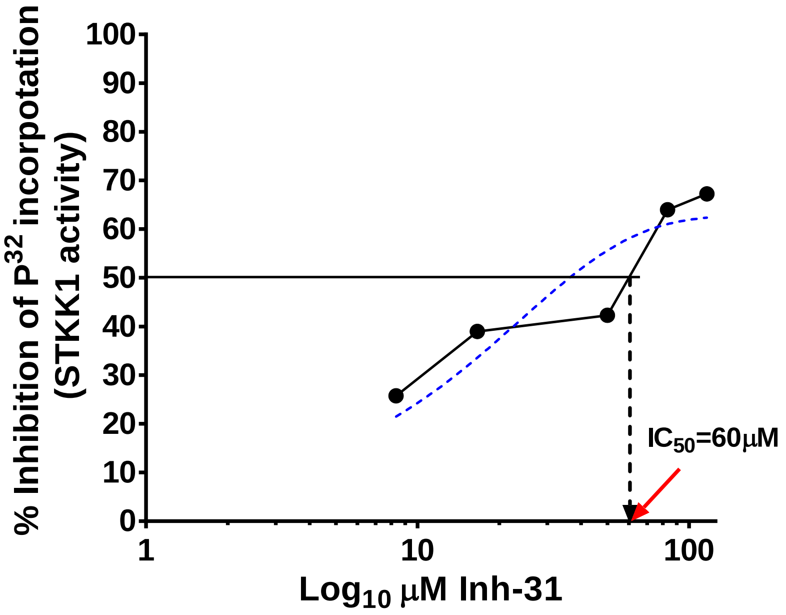

B

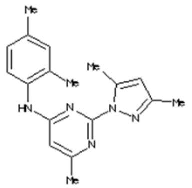

N-(2,4-dimethylphenyl)-2(3,5-dimethylpyrazol-1-yl)-6-methyl pyrimidine -4 amine

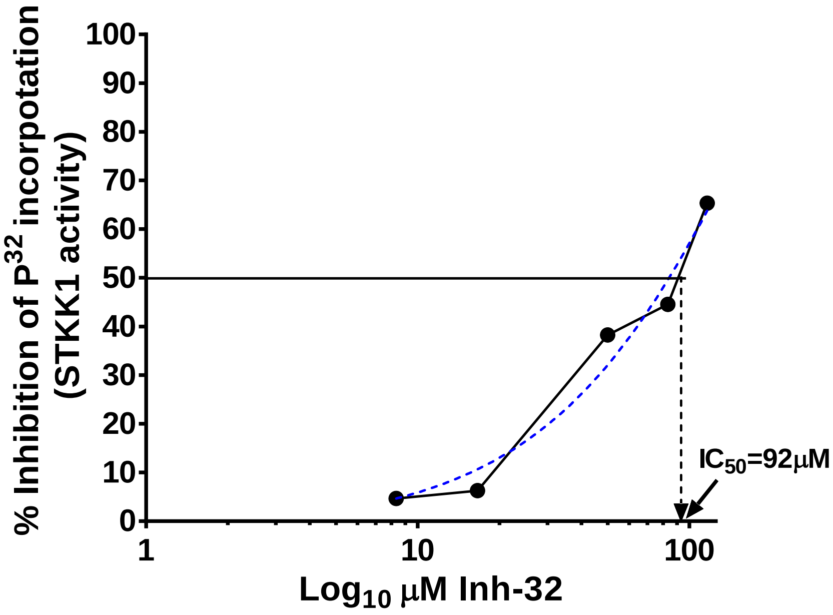

Fig. S4. Determination of IC_{50} concentrations of Inh-31 (A) and Inh-32 (B). Dotted lines depicts regression line. Each data point represents an average reading obtained from two independent experiments.

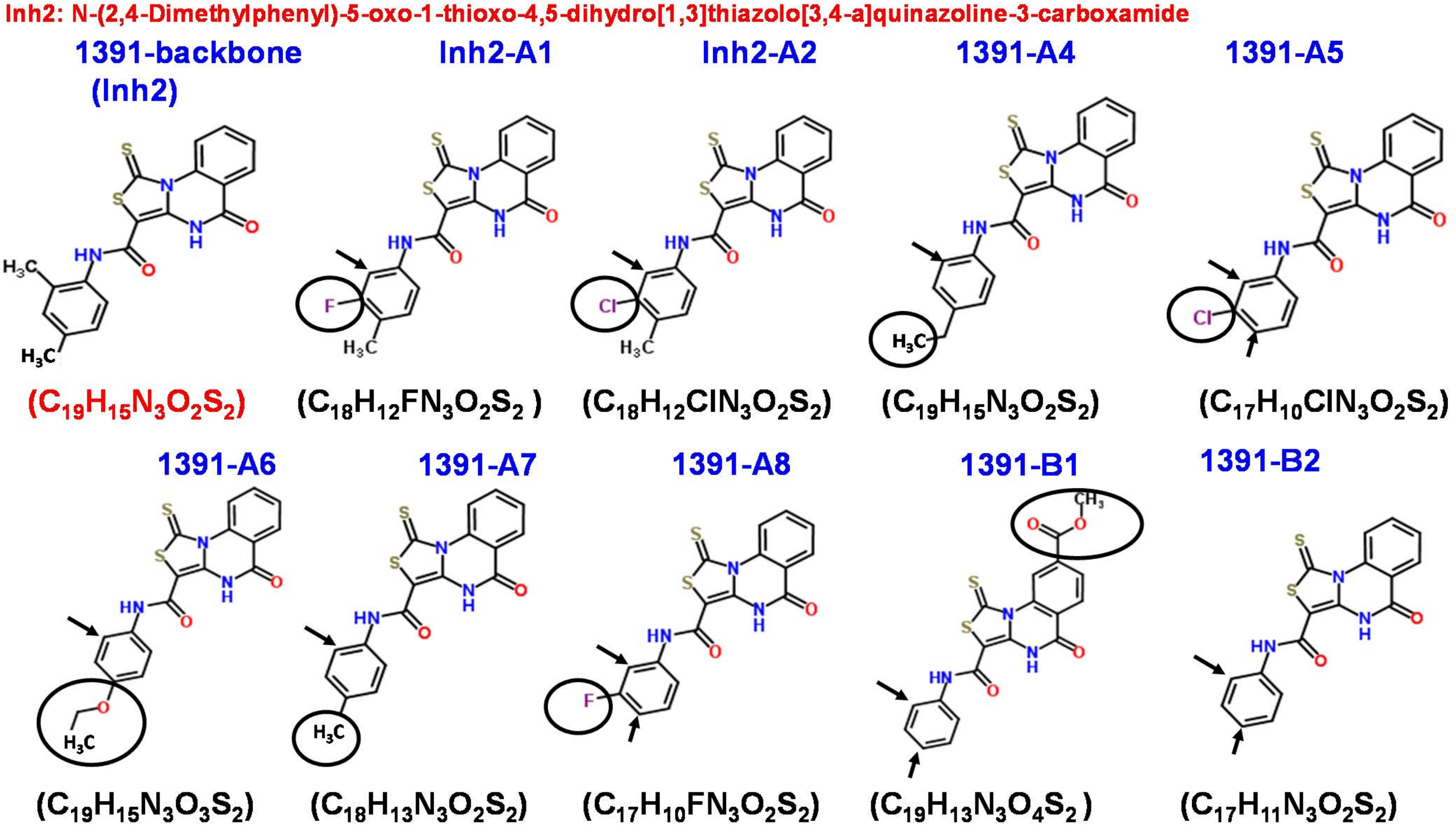

Figure S5. Structure of Inh-2 (compound 1391) and its derivatives. Circled area denotes the location of the side chain modification. The arrow denotes the site of the original side chain that was deleted to modify the Inh2 and derive new structures.

Compounds	Structure	Binding Energy (kcal/mol)	No. of Conformers
ANP	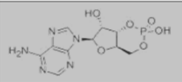	-9.53	46
Inh2-lead	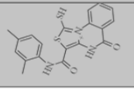	-10.04	68
A1	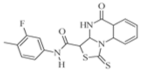	-9.86	31
A2	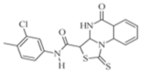	-10.36	77
A4	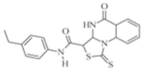	-10.23	41
A5	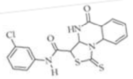	-9.93	89
A6	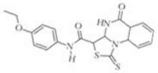	-9.89	37
A7	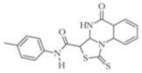	-10.80	66
A8	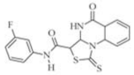	-9.53	28
B1	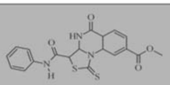	-11.58	21
B2	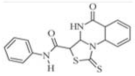	-8.78	32

Figure-S6 Comparison of molecular docking analysis-based binding affinities of Inh-2 and its derivatives with crystal structure of the kinase domain of *S. aureus* STK1 (PDB ID 4EQM).

A

Inh2-B1 (µM) is listed down the rows. Ceftriaxone concentrations are listed across the columns.

	Inh2-B1 (µM)	1	2	3	4	5	6	7	8	9	10	11	12
Ceftriaxone (µM)			151	75.5	37.8	18.9	9.4	4.7	2.36	1.18	0.59	0.3	0.15
Ceftriaxone (µg/ml)			100	50	25	12.5	6.3	3.1	1.6	0.78	0.4	0.2	0.1
A		23±3**	0	12	22	22	21	20	22	20	20	19	20
B	100	0	0	0	0	0	0	0	0	0	0	0	0
C	50	7	0	0	0	0	0	0	0	0	0	1	3
D	25	16	0	6*	10	14	17	15	14	15	14	16	13
E	12.5	22	0	14*	17	17	17	20	20	22	20	21	22
F	6.25	20	0	15*	19	20	20	20	20	20	20	20	18
G	3.125	19	0	12*	22*	22	22	22	20	20	22	18	16
H	1.56	20	0	9*	19*	20	20	20	20	18	16	18	18

B

Inh2-B1 (µM) is listed down the rows. Cefotaxime concentrations are listed across the columns.

	Inh2-B1 (µM)	1	2	3	4	5	6	7	8	9	10	11	12
Cefotaxime (µM)			220	110	55	27.5	13.8	6.9	3.4	1.7	0.86	0.43	0.21
Cefotaxime (µg/ml)			100	50	25	12.5	6.3	3.1	1.6	0.78	0.4	0.2	0.1
A		21±4**	0	11	16	21	22	18	25	22	21	21	20
B	100	0	0	0	0	0	0	0	0	0	0	02	02
C	50	9	0	0	0	0	01	07	07	06	5	5	12
D	25	12	0	5*	15	14	15	19	13	17	13	16	17
E	12.5	20	0	14	14	15	20	20	20	20	18	20	21
F	6.25	24	0	17	20	20	18	20	20	20	19	20	23
G	3.125	18	0	17	20	20	20	20	18	17	13	21	20
H	1.56	15	0	15*	20	20	15	16	17	15	15	15	15

Fig. S7 Checkerboard assay for determining bactericidal assay. A serial two-fold dilutions of 2mM of Inh2-B1 (MW- 411.58) and (A) 3mM of Ceftriaxone (MW 669.7) were made in two separate 96-well plates in vertical and (B1-B12 to H1-H12 wells) and horizontal (A2-H2 to A12-H12 wells) directions respectively. Serially diluted Inh2-B1 (100µl) in the individual well was then mixed with the 100 µl serially diluted ceftriaxone present in the corresponding wells. The resulting various combinations of Inh2-B1(starting from 1mM) and Ceftriaxone (starting from 1.5 mM) or Cefotaxime (2.1mM) as well as serially diluted only Inh2-B1(B1 to H1) and ceftriaxone (A2-A12) or cefotaxime (A2-A12) were then incorporated in Muller-Hinton mini agar plates (2ml) . The agar plates were then seeded with diluted *S. aureus* MW2-wildtype culture equivalent to approximately 23±3 CFU. This checkerboard assay was used to determine the ideal combination of lowest concentration Inh2-B1 and Ceftriaxone that kills 99.9% of the wild-type MW2. (B) a similar procedure as described above was employed to determine the impact on the bactericidal activity of Cefotaxime in the presence or absence of Inh2-B1.

** CFU colony forming units of *S. aureus* MW2- A1 well shows CFUs without Inh2-B1 and Ceftriaxone. A2- A12 CFUs only in the presence of Ceftriaxone/ Cefotaxime . B1-H1 CFUs only in the presence of Inh2-B1. * Minute colonies appeared after 48 h incubation.

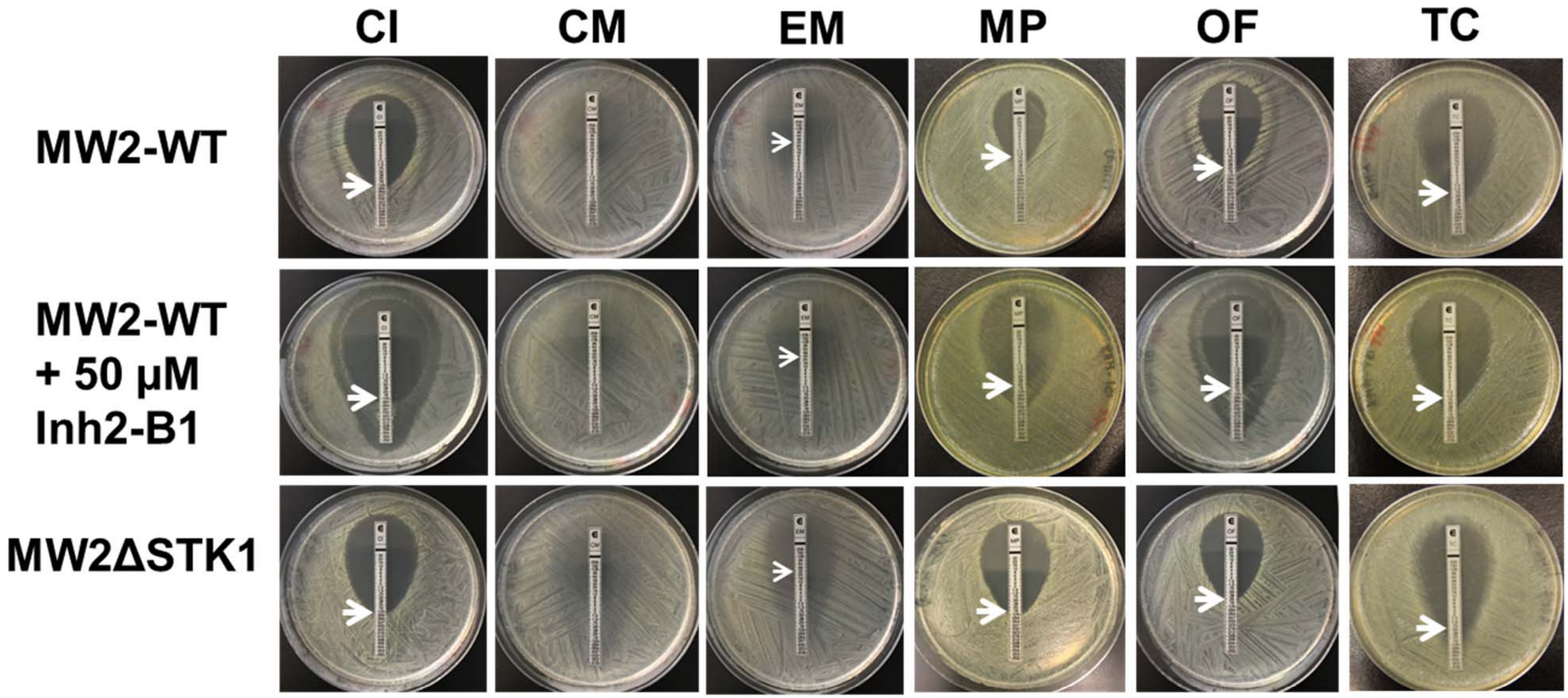

Fig. S8. **Susceptibility patterns of S. aureus MW2-WT and S. aureus MW2DSTK1 against various antibiotics as determined by E-test™.** Antibiotic susceptibility of MW2-WT strain was measured in the presence and absence of 50 µM Inh2-B1 and their comparison with S. aureus MW2DSTK1 mutant strain carried out in the absence of Inh2-B1. White arrows indicate MIC values (µg/ml) shown underneath each panel of antibiotic. CI- Ciprofloxacin, CM-Clindamycin, EM-erythromycin, MP-Meropenem, OF-Ofloxacin, TC-tetracyclin

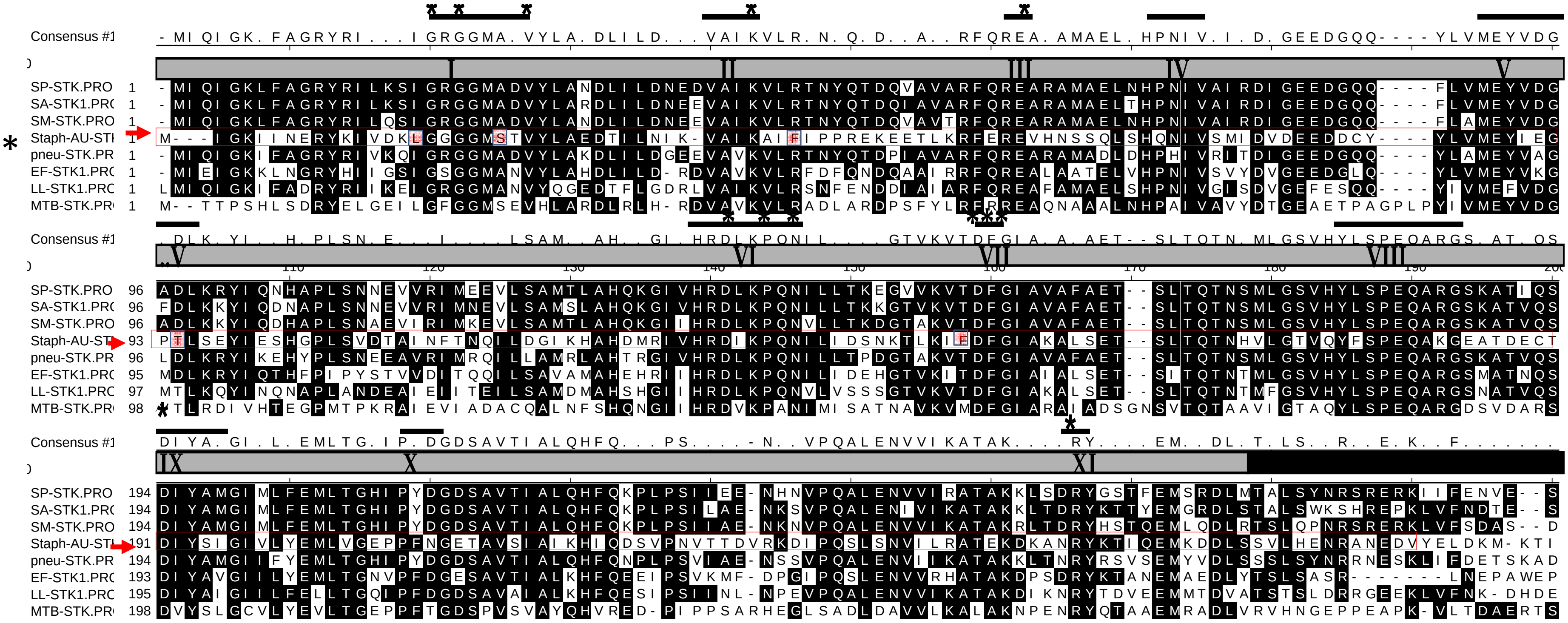

Fig . S9. Comparison of the amino acid sequence of kinase domain of S. aureus STK1 with those of other gram-positive bacteria and *M. tuberculosis*. The boxed sequence (*) in red denotes *S. aureus* STKK1 (Staph-AU-Stk1). The STKK1 in the present study included 280 aa. Sequence in black denote the conserved residues. Hank's motif (I-XI) region for catalytic kinase activity is shown above the sequence comparison. Note the P-loop $L^{16}G^{17}GGG^{20}$ in *S. aureus* STKK1 instead of a conserved GRGGMAD motif. S^{22},F^{37},T^{94} and F^{150} (pink box) are the unique residues of the catalytic domain *of S. aureus* STKK1. Other Ser/Thr Kinases shown in the comparison are from *S. pyogenes* (SP-STK), *S. agalactiae* (SA-STK1), *S. mutans* (SM-STK), *S. pneumoniae* (Pneu-SATK), *Enterococcus faecalis* (EF-STK), *Listeria sp* (LL-STK1) and *M. tuberculosis* (MTB-STK).
